# Challenges and strategies for managing early-to-moderate chronic kidney disease in primary care: A systematic integrative review in high-income countries

**DOI:** 10.1080/13814788.2026.2704287

**Published:** 2026-08-05

**Authors:** Agnès Oude Engberink, Elodie Million, Killian L’Helgouarc’h, Alix Tarbé, Armel Leroux, Fanny Jourdain, Béatrice Lognos, Olivier Moranne

**Affiliations:** a Desbrest Institute of Epidemiology and Public Health, UMR UA1368 INSERM – University of Montpellier, Montpellier, Occitanie, France; b Department of General Medicine, Faculty of Medicine, University of Montpellier, Montpellier, Occitanie, France; c Nephrology-Dialysis-Apheresis Department, Carémeau University Hospital, Nîmes, Occitanie, France

**Keywords:** Primary care, chronic kidney disease, care pathway, early detection, guideline adherence

## Abstract

**Background:**

Early-to-moderate chronic kidney disease (CKD) is largely managed in primary care, yet reported practices vary across settings. Understanding how CKD is identified and managed in routine practice is essential to inform improvements in care.

**Objective:**

To synthesise empirical evidence on the management of adults with early-to-moderate CKD in primary care in high-income countries including real-world practices and initiatives to improve CKD management.

**Methods:**

We conducted a systematic integrative review of quantitative, qualitative, and mixed-methods studies published between 2000 and 2022. MEDLINE, Embase, the Cochrane Library, KT+, and PDQ-Evidence databases were searched. Eligible studies reported management practices for early-to-moderate CKD in primary care. Findings were synthesised using a convergent integrated narrative approach.

**Results:**

A total of 90 studies were included (46 descriptive, 23 qualitative or mixed-methods studies, and 21 interventional), representing over 33 million patients and 3,180 healthcare professionals. Across studies, substantial variability in screening, monitoring, referral practices, and implementation of management strategies was reported. Low screening rates among at-risk populations, incomplete follow-up after abnormal results, variability in medication use and risk-factor control, and heterogeneous referral practices were common findings. Patients often had limited awareness of CKD, and organisational barriers affected engagement in care. Various initiatives, including interprofessional models, decision-support tools, patient education, and multimodal interventions, showed heterogeneous effects across settings.

**Conclusions:**

Management of early-to-moderate CKD in primary care varies considerably across high-income countries. Reported findings suggest that improving CKD care may require context-adapted strategies combining organisational, clinical, and educational components, particularly those involving multidisciplinary collaboration and decision support.

## KEY MESSAGES

Early-to-moderate CKD remains underdetected and suboptimally managed in primary care in high-income countries.

Limited CKD knowledge, poor guideline adherence, and fragmented coordination hinder effective management and timely referral.

Multimodal, team-based interventions combining digital tools, nurse coordination, and patient education offer the most promising improvements.

## Introduction

More than 850 million people worldwide (9% of the global population) are affected by chronic kidney disease (CKD) [1]. Effective management of CKD patients requires coordinated care models and personalised care pathways involving multiple healthcare providers [2]. CKD is associated with increased cardiovascular risk, with an 8 to 10-fold increase in mortality among patients with diabetes or hypertension [3,4]. The staging classification introduced in 2002 [5] and has defined by international guidelines in 2012 [6] defines stage-specific strategies ranging from early detection to kidney-protective measures, prevention of complications, referral to nephrology, and preparation for kidney replacement therapy. Despite clear recommendations, early-stage CKD remains frequently underdiagnosed [7], although identifying at-risk individuals improves outcomes and reduces healthcare costs [8,9]. Primary care plays a key role in early CKD detection, with guidelines recommending creatinine and albuminuria testing, particularly the Albumin-to-Creatinine Ratio (ACR) [10]. Screening is crucial in patients with cardiovascular risk [11,12], especially given the proven benefits of antiproteinuric therapies [13,14]. Primary care clinicians, including general practitioners, nurses, and nurse practitioners (NPs), play a central role in early CKD management [15,16], but co-management with nephrologists is often limited [17]. CKD care pathways are complex and vary widely in design and implementation [18–20]. KDIGO therefore advocates transitioning from a binary pre/post-referral model towards a continuum of shared management involving patients, caregivers, and healthcare professionals [21]. Interprofessional teams can deliver care adapted to local needs and patient complexity, yet substantial gaps remain between guidelines and practice [22–24], partly due to CKD heterogeneity and the need to better identify patients at risk of progression [9]. A recent *Lancet* editorial highlighted the WHO resolution urging investment in CKD prevention and early care [25]. Because CKD management and care pathways are closely interdependent, this review uses the term ‘care management’ to encompass both. This systematic integrative review aimed to synthesise empirical evidence on the management of outpatients with early-to-moderate CKD in primary care settings in high-income countries, including reported practices, challenges, and initiatives to improve care management.

By integrating findings from heterogeneous study designs, this review provides a comprehensive overview of real-world CKD care and identifies gaps between recommendations and routine practice.

## Methods

### Study design

This systematic review adopts an integrative evidence synthesis approach, as described in the JBI Manual for Evidence Synthesis [26], allowing the inclusion of diverse empirical study designs. The review was conducted in accordance with PRISMA guidelines [27] (see Table S1) and prospectively registered in PROSPERO (CRD42022314490).

We aimed to synthesise empirical evidence on how early-to-moderate CKD is managed in primary care among adults in high-income countries, including reported practices, challenges, and initiatives to improve care management.

### Setting & study population

We focused on adults (≥18 years) with early-to-moderate CKD managed in outpatient settings in high-income countries. Relevant care settings included primary care, community care, and ambulatory nephrology clinics. Eligible healthcare providers included general practitioners, nurses or nurse practitioners, pharmacists and relevant medical specialists involved in CKD management.

### Selection criteria for studies

We included primary studies reporting outpatient management of adults (≥18 years) with CKD and an estimated glomerular filtration rate (eGFR) >45 ml/min/1.73 m^2^ [6]. Studies including patients with lower eGFR values were retained only when most participants met inclusion criteria. We excluded studies on pregnancy, advanced CKD, dialysis, transplantation, laboratory-based analyses, caregivers, case reports, reviews, meta-analyses and editorials.

### Information sources and search strategy

We searched MEDLINE, Embase, Cochrane Library, Knowledge Translation+ (KT+) [28], and PDQ-Evidence [29] for studies published in English between 1 January 2000 and 31 December 2022. This period corresponds to the introduction and consolidation of contemporary CKD definitions and staging systems following the 2002 KDOQI guidelines. KT+ and PDQ-Evidence databases description are available in Table S2. Only scientific publications conducted in high-income countries, as defined by the 2022 World Bank Atlas [30], were included. Search strategies were developed by a multidisciplinary team (GPs, nephrologist, trainee, patient partner), using the PESICO framework [31] and MeSH terms/keywords, refined with support from a university librarian. Full strategies are provided in Table S3. Reference lists of included articles were screened manually to identify additional relevant studies.

### Study selection

Search results were imported into Rayyan [32]. Titles and abstracts were screened independently by two reviewers; disagreements were resolved by a third reviewer. For multiple reports of the same study, the most complete version was retained.

### Data extraction

Data extracted included study characteristics (country, design, sample, population), objectives, methods, outcomes, results, and limitations. Two reviewers using a standardised form performed extraction independently. Because included studies used heterogeneous designs, all reported measures and findings related to CKD management practices, processes of care, or implementation were extracted.

### Risk of bias assessment

Risk of bias was assessed independently by at least two reviewers using the Joanna Briggs Institute (JBI) tools appropriate to each study design [33], with the first author participating in all assessments. Disagreements were resolved by consensus, with adjudication by a senior author when required. Studies with an average score <5/10 were excluded. Details of JBI scoring are provided in Table S4.

### Analytical approach

Given the heterogeneity of study designs, populations, and outcomes, results were synthesised narratively following guidance for integrative evidence synthesis as described by the Joanna Briggs Institute [26,34]. Consistent with this approach, no single predefined outcome measure was required for inclusion.

During study identification and extraction, the PESICO framework informed broad outcome areas of interest and findings were initially organised within six broad domains of CKD care management (screening and detection, CKD identification and coding, kidney-protective management and follow-up, referral and collaboration with nephrology, patient education and self-management, and organisation of care pathways). These domains served solely as a descriptive framework for organising and mapping heterogeneous evidence and were not analytical categories.

Subsequently, an inductive integrative narrative synthesis was conducted to identify overarching themes and subthemes across studies. No themes or subthemes were predefined. Rather, themes emerged iteratively through the synthesis process. Emergent themes frequently spanned several domains and provided a more nuanced understanding of real-world CKD management than broad descriptive categories alone.

### Patient and public involvement

A patient partner contributed to the design of the search strategy, study selection, and interpretation of findings.

## Results

### Study selection and characteristics

**Figure 1.**
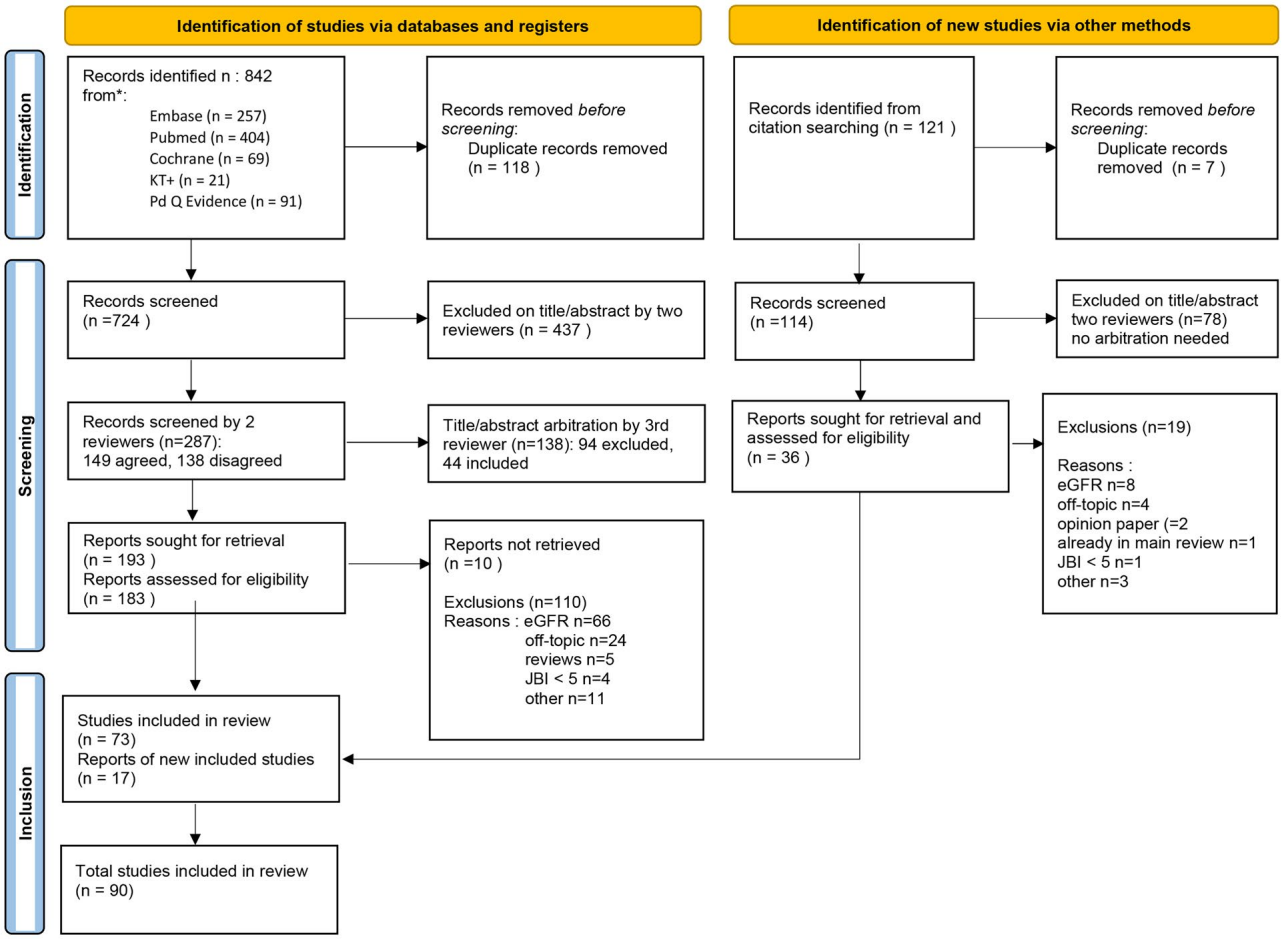
PRISMA flow diagram of study selection. Flow diagram describing identification, screening, eligibility assessment, and inclusion of studies following PRISMA guidelines.

Figure 1 presents the PRISMA flow diagram. Studies were categorised as descriptive (D1–D46) [22,35–79], qualitative or mixed-methods (Q47–Q69) [15,17,23,80–99], and interventional (I70–I90) [100–120]. Tables 1–3 summarise the included descriptive (D), qualitative/mixed-methods (Q), and interventional (I) studies and provide their corresponding study codes, authors and references. Among descriptive studies, 21 were cross-sectional, 20 cohort (17 retrospective, 3 prospective), and 5 other designs. Of the qualitative studies, 5 used mixed methods. Among interventional studies, 15 were randomised controlled trials. Overall, data from 33,470,926 patients (over 28 million from D22 [56]) and 3,180 healthcare professionals were included, 2,688 general practitioners, 352 nurses or nurse practitioners, and 60 nephrologists. Study characteristics, including geographical distribution, publication years and participant characteristics, are detailed in Tables S5–S8. Domains of CKD care management addressed across studies are summarised in Table S10. Detailed extracted data for each included study are provided in Tables S11–S13.

**Table 1.** Summary of descriptive studies (D1–D46).

Code	First author, Year, [ref]	Continent, Country	Type of study	Participants : Patients, GPs, Nurses, APNs, nephrologists	JBI
D1	Van Dipten, 2017, [35]	Europe, Netherlands	Prospective cohort study	2772 patients	7
D2	Fox, 2008, [36]	America, USA	Prospective cohort study	181 patients, 6 GPs, 4 NPs	6.7
D3	Tan J, 2014, [37]	Oceania, New Zealand	Prospective cohort study	47 patients	6.9
D4	Danforth KN, 2019, [38]	America, USA	Prospective cohort study	244,540 patients, 15 GPs	8.4
D5	Jain P, 2014, [39]	Europe, UK	Retrospective cohort study	2,707,130 patients	8.2
D6	Donald M, 2021, [40]	America, Canada	Retrospective cohort study	345,058 patients	8.9
D7	Bansal S, 2020, [41]	America, USA	Retrospective cohort study	270,170 patients	8.5
D8	Nash D.M, 2017, [42]	America, Canada	Retrospective cohort study	184,557 patients	7.1
D9	Chen Y-C, 2020, [43]	Asia Taiwan	Retrospective cohort study	173,162 patients	8.4
D10	Bramlage P, 2021, [44]	Europe, Germany	Retrospective cohort study	134,395 patients	7.8
D11	James G, 2022, [45]	America, Europe USA UK	Retrospective cohort study	123,807 patients	8.3
D12	Chong C, 2022, [46]	America, Canada	Retrospective cohort study	86,475 patients	10
D13	Ghimire A, 2022, [47]	America, Canada	Retrospective cohort study	69,372 patients	8.1
D14	Singh K, 2017, [48]	America, USA	Retrospective cohort study	56,461 patients	8.9
D15	Kampmann J.D, 2022, [49]	Europe, Denmark	Retrospective cohort study	19,743 patients	7.9
D16	Van Gelder V, 2016, [50]	Europe Netherlands	Retrospective cohort study	8794 patients	8.6
D17	Jones R.K, 2013, [51]	Europe UK	Retrospective cohort study	2710 patients	8.2
D18	Perkins R M, 2016, [52]	America, USA	Retrospective cohort study	2472 patients	7.5
D19	Weckmann G, 2022, [53]	Europe, Germany	Retrospective cohort study	1778 patients	8.6
D20	Frigaard M, 2019, [54]	America, USA	Retrospective cohort study	1767 patients	9.2
D21	Van Dipten C, 2021, [55]	Europe, Netherlands	Retrospective cohort study	300 patients	9.7
D22	Alfego D, 2021, [56]	America, USA	Cross sectional study	28,295,982 patients	7.8
D23	Chu Chi D, 2022 [57]	America USA	Cross-sectional study	291,155 patients	10
D24	Manski-Nankervis JA, 2018, [58]	Oceania, Australia	Cross-sectional study	90,550 patients	7.5
D25	Bello A.K, 2019, [59]	America, Canada	Cross-sectional study	46,162 patients	8.1
D26	Bezabhe, W.M, 2020, [60]	Oceania, Australia	Cross-sectional study	44,259 patients	8.6
D27	Hagnäs M, 2020, [61]	Europe, Finlande	Cross-sectional study	5112 patients	7.5
D28	Samal L, 2014, [62]	America, USA	Cross-sectional study	3118 patients	10
D29	Samal L, 2015, [63]	America, USA	Cross-sectional study	3118 patients	9.7
D30	Koraishy F.M, 2017, [64]	America, USA	Cross-sectional study	2170 patients	9.4
D31	McIntyre N.J, 2010, [65]	Europe, UK	Cross-sectional study	1741 patients	7.8
D32	Schulz C, 2022, [22]	Europe, France	Cross-sectional study	1547 patients	9.8
D33	Gaffney H, 2014, [66]	Europe, UK	Cross-sectional study	436 patients	9
D34	Greer RC, 2011, [67]	America, USA	Cross-sectional study	236 patients, 40 GPs	7.5
D35	Burke, M. T, 2014, [68]	Oceania, Australia	Cross-sectional study	210 patients	5.7
D36	Scherpbier-de Haan ND, 2013, [69]	Europe, Netherlands	Cross-sectional study	122 patients	7.2
D37	Meran S, 2011, [70]	Europe, UK	Cross-sectional study	88 patients	7.2
D38	Tatematsu S, 2022, [71]	Asia, Japan	Cross-sectional study	1148 GPs	8
D39	Tatematsu S, 2021, [72]	Asia, Japan	Cross-sectional study	585 GPs	8.4
D40	Abdel-Kader K, 2014, [73]	America, USA	Cross-sectional study	165 GPs	7.8
D41	Seidu S, 2022, [74]	Europe, Belgium, Italy, UK, Spain, Turkey	Cross-sectional study	220 GPs,46 nurses	7.8
D42	Pang J, 2016, [75]	America, Canada	Cross-sectional study	89 GPs, 7 nephrologists	7.2
D43	Lee B, 2012, [76]	America, USA	Case-control study	5916 patients	9
D44	Escribá-Martí G, 2022, [77]	Europe, Spain	Feasability study	198 patients	6.9
D45	Jameson K, 2014, [78]	Europe, UK	Prevalence study	165,942 patients	8.6
D46	Smith Z.G, 2016, [79]	America, USA	Prevalence study	1255 patients	8.1

**Table 2.** Summary of qualitative and mixed-methods studies (Q47–Q69).

Code	First author, Year, [ref]	Continent, Country	Type of study	Participants	JBI
Q47	Vest B, 2015, [80]	America, USA	Qualitative study	24 GPs, 3 nurses or PHP assistants	9,5
Q48	Havas K, 2017, [81]	Oceania, Australia	Qualitative study	63 patients	9
Q49	Kahn L, 2015, [82]	America, USA	Qualitative study	33 patients	8,7
Q50	van Dipten C, 2018, [83]	Europe, Netherlands	Qualitative study	21 patients	8,5
Q51	Hwang SJ, 2020, [84]	Asia, Singapour	Qualitative study	20 patients	9
Q52	Sinclair P, 2017, [85]	Oceania, Australia	Qualitative study	26 practice nurses	5.7
Q53	Greer RC, 2012, [86]	America, USA	Qualitative study	18 GPs	7.5
Q54	Van Dipten C, 2018, [87]	Europe, Netherlands	Qualitative study	27 GPs	9.8
Q55	Lo C, 2016, [88]	Oceania, Australia	Qualitative study	65 professionals	9.7
Q56	Greer R.C, 2019 [17]	America, USA	Qualitative study	13 GPs, 19 GP trainees	7.7
Q57	Blakeman T, 2012, [89]	Europe, UK	Qualitative study	11 GPs 10 nurses	8
Q58	Gulla J, 2017, [90]	America, USA	Qualitative study	12 GPs	7
Q59	Thomas N, 2019, [91]	Europe, UK	Qualitative study	6 GPs, 1 pharmacist, 1 coordinator	7,5
Q60	Crinson I, 2010, [92]	Europe, UK	Qualitative study	26 GPs, 9 APNs 1 pharmacist	7.5
Q61	Nihat AS, 2016, [15]	Europe, UK	Qualitative study	72 to 117 primary healthcare professionals	8.5
Q62	McBride D, 2014, [93]	America, USA	Qualitative study	19 GPs, 1 APN	7
Q63	Simmonds R, 2016, [94]	Europe, UK	Qualitative study	16 GPs, 3 Infirmier APNs, 4 nephrologists, 2 public health physicians	7.5
Q64	Haase A, 2019, [95]	Europe, Germany	Qualitative study	20 nephrologists	6.5
Q65	Rainey H, 2020, [96]	Europe, UK	Mixed-methods study	9 patients	6.5
Q66	Donald M, 2016, [97]	America, Canada	Mixed-methods study	10 GPs, 3 APNs, 3 nephrologists, 2 pharmacists	7.5
Q67	Lo C, 2016, [98]	Oceania, Australia	Mixed-methods study	22 GPs	7
Q68	Haley WE, 2015, [99]	America, USA	Mixed-methods study	48 GPs, 16 nephrologists	7.7
Q69	Sperati C.J, 2019, [23]	America USA	Mixed-methods study	32 GPs	8.7

**Table 3.** Summary of interventional studies (I70–I90).

Code	First author, Year, [ref]	Continent, Country	Type of study	Participants	JBI
I70	Major RW, 2019, [100]	Europe, UK	Randomised controlled trial (RCT)	23 357 patients	6,9
I71	Sequist TD, 2018, [101]	America, USA	RCT	7 691 patients, 153 GPs	7,9
I72	Carroll JK, 2018, [101]	America, USA	RCT	6 699 patients	8,5
I73	van Gelder V, 2017, [103]	Europe, Netherlands	RCT	3004 patients	8,2
I74	Yamagata K, 2016, [104]	Asia, Japan	RCT	2 379 patients	8.9
I75	Drawz PE, 2012, [105]	America, USA	RCT	781 patients, 8GPs, 6 APNs, 56 trainee	7.7
I76	Tuot D.S, 2018, [106]	America, USA	RCT	746 patients, 80GPs, 16 nurses /APNs	7.9
I77	Peralta C.A, 2020, [107]	America, USA	RCT	524 patients	7.3
I78	Blakeman T, 2014, [108]	Europe, UK	RCT	436 patients	6.4
I79	Al Hamarneh, 2018, [109]	America, Canada	RCT	290 patients	8.1
I80	Roddy MK, 2022, [110]	America, USA	RCT	271 patients	7.5
I81	Scherpbier-de Haan, 2013, [111]	Europe, Netherlands	RCT	164 patients	8.5
I82	Walker RC, 2014, [112]	Oceania, New Zealand	RCT	52 patients	10
I83	Chang AR, 2016, [113]	America, USA	RCT	47 patients	7.5
I84	Sinclair PM, 2019, [114]	Oceania, Australia	RCT	212 GPs	9.2
I85	Pesce F, 2022, [115]	Europe, Italy	Proof-of-concept study	18 662 patients, 17GPs 2 nephrologists	8.5
I86	Akbari A, 2012, [116]	America, Canada	Quality improvement study	2 672 patients	8.7
I87	Vassalotti J, 2019, [117]	America, USA	Quality improvement study	7 420 patients	8.1
I88	Chiu Chu L, 2013, [118]	Asia, Taiwan	Before -and-after pilot study	33 patients	6.9
I89	Stoves J, 2010, [119]	Europe, UK	Mixed-methods study	466 patients, 16 GPs, 2 nephrologists	7.6
I90	Havas K, 2018, [120]	Oceania, Australia	Before-and- after study	78 patients	8.1

### Summary of findings

**Figure 2.**
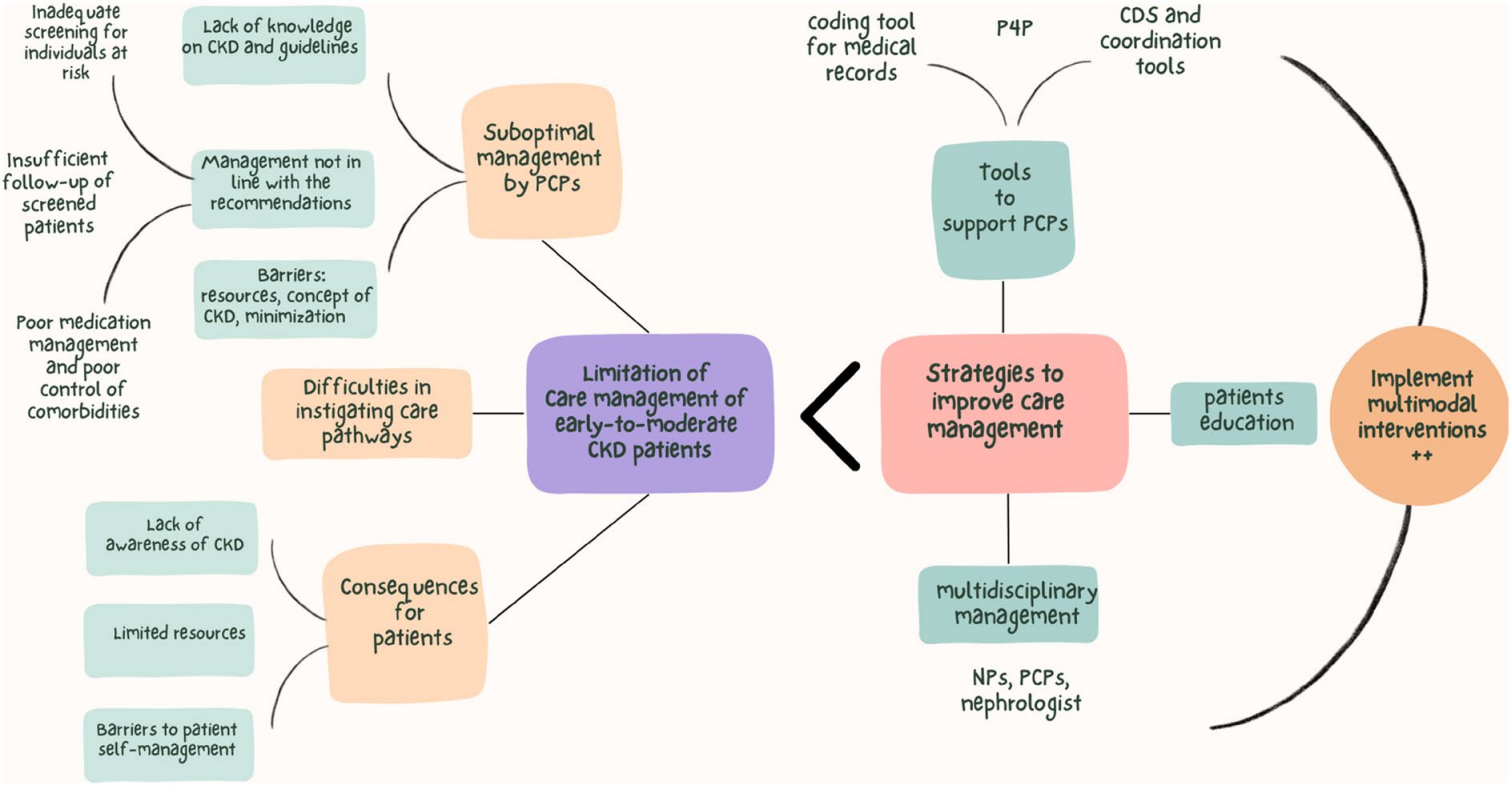
Conceptual model for optimising early-to-moderate CKD care. Model illustrating current care gaps and potential strategies to improve CKD management in primary care.

Overarching themes and subthemes emerging from the integrative synthesis are summarised in Table 4. A detailed synthesis of initiatives to improve CKD management is presented in Table 5. Figure 2 presents a conceptual model of current CKD management and potential improvement strategies.

**Table 4.** Summary of themes and subthemes across included studies.

Overarching themes	Emergent subthemes
Early-to-moderate CKD management in primary care	Reported gaps in CKD management in primary care
	Referral practices and organisation of care pathways
	Consequences for patients
	Professionals’ needs to improve CKD management
Initiatives to improve CKD management	Training interventions for healthcare professionals
	Organised screening campaigns
	Interprofessional management models
	Tools to support primary healthcare professionals
	Patient education and self management interventions
	Multimodal interventions combining human support and tools

Themes and subthemes emerged inductively through the integrative narrative synthesis of heterogeneous evidence and were not predefined analytical categories.

**Table 5.** Themes and subthemes related to initiatives to improve CKD management in primary care.

Initiatives to improve the management of early-to-moderate stage CKD patients		
Effects of training interventions for healthcare professionals		I84, I87, Q61
Organised screening campaigns		D9
Multiprofessional management models	*Pharmacist-led interventions*	I77, I79, I83, D44, D46
	*Role of nephrology-trained NPs in supporting PCPs*	I70, I82
	*Support from nephrologists and tele-expertise*	D36, D42, D43, I73, I85, I89
Tools to support primary care professionals	*Identification through coding and CKD registers*	*D5, D7, D20, D28, I75, Q61*
	*Pay-for-performance schemes based on quality indicators*	*D8, D25, D45, Q61*
	*Clinical decision-making support and care coordination tools*	*Q59, Q66, Q68, D6*
Patients education interventions		*I74,I78, I80, I88, I90, Q48*
Multimodal interventions		*I72, I71 I81, D2, D3, D45*

Themes and subthemes emerged inductively through the integrative narrative synthesis of heterogeneous evidence and were not predefined analytical categories.

#### Identification and management of early-to-moderate CKD patients

##### Reported gaps in CKD management in primary care

Across studies, authors consistently reported gaps in CKD management in primary care.

###### Low screening rates in at-risk populations

Large population-based studies reported low screening rates among at-risk populations. In the USA, up to 80% of at-risk individuals were not appropriately screened for CKD (D22, D23), with similar findings reported in Australia, Canada, Germany, Finland, and the UK (D24, D8, D10, D19, D27, D11). Although urine testing increased in some contexts, proteinuria assessment and risk stratification remained insufficient (D7, D18).

###### Incomplete follow-up and monitoring after abnormal results

Abnormal eGFR values often did not trigger repeat testing or structured follow-up (D4, D16). In Canada, incomplete monitoring of creatinine and potassium after RASi initiation was repeatedly observed (D8, D25).

###### Variability in medication use and risk factor control

Descriptive studies highlighted underuse of recommended therapies and inappropriate prescribing. While RASi were widely prescribed in Canada, statin use remained insufficient (D8, D25). In Germany and Finland, only about half of diabetic-hypertensive patients received RASi (D10, D27). Exposure to potentially nephrotoxic medications remained frequent, particularly in Australia (D26), and uptake of SGLT2 inhibitors and GLP-1 agonists was very low (D27). Control of blood pressure and glycaemia was often incomplete in several European cohorts (D10, D16, D31).

###### Obstacles reported by healthcare professionals

Professionals reported time constraints, information overload, and limited EHR usability as major barriers (Q53, Q69, D4). Qualitative studies described diagnostic uncertainty, ethical concerns around CKD disclosure, and reluctance to label CKD – particularly in older adults – due to fears of causing anxiety or overmedicalisation (Q51, Q52, Q54, Q57, Q60, Q63). Some GPs preferred alternative terms to ‘CKD’ (Q57), while interprofessional communication gaps and lack of educational resources further limited effective therapeutic education (Q51, Q64).

##### Referral practices and organisation of care pathways

Referral practices varied substantially across studies. Although automated eGFR reporting increased nephrology referrals (I86), a proportion of referrals did not meet guideline criteria (D13). Conversely, many patients meeting referral criteria, including those with advanced CKD or rapid eGFR decline, were not referred (D1, D23, D14, D30). Differences between referral criteria across guidelines (HAS, KDIGO, KFRE) resulted in substantial variation in which patients met referral thresholds (D32). Several studies reported mismatches between patient needs and care location: stable patients were often followed in nephrology, whereas those with progressive CKD were less frequently referred (D17). Between 23% and 30% of nephrology patients could be safely redirected to primary care, with stable or improved eGFR (D21, D37). While co-management with nephrologists improved screening and prescribing at stage 3 CKD (D29), limited coordination, unclear role attribution, and insufficient interoperability were associated with follow-up losses and redundant testing (D4, Q56, Q60, D19, Q64).

##### Consequences for patients

Many patients were unaware of their diagnosis or its implications (Q53, Q49, Q47, Q69, D34, D35, D33, D31, D19). Asymptomatic disease, communication challenges, and misconceptions were reported as barriers to engagement and adherence (Q49, Q50, Q65, D35). Access barriers, including time, travel distance, and limited coverage, were also reported to affect self-management (Q49, Q47, Q69, D4). While diagnosis could generate anxiety, patient-centred communication and family involvement were reported to support behavioural change (Q51, Q65).

##### Professionals’ needs to improve CKD management

PCPs and nephrology leaders highlighted the need for clearer quality indicators, improved EHR usability, and better clinical decision support (Q47, Q55, D4). Desired tools included clearer visualisation of eGFR trends, alerts for risk factors, improved coordination tools, and access to nephrology data to support risk-based patient prioritisation (Q55, Q58, Q62, Q69).

#### Initiatives to improve the management of patients with early-to-moderate CKD

##### Effects of training interventions for healthcare professionals

Training interventions for healthcare professionals showed variable effects across studies. In Australia, a behavioural intention–based programme for primary care nurses did not improve CKD screening compared with a knowledge-based approach (I84). In the USA, guideline reminders modestly increased uACR and eGFR testing but did not change prescribing (I87). PCPs nevertheless perceived specialist-led audits as more useful than passive information delivery (Q61).

##### Organised screening campaigns

Large-scale screening increased detection and follow-up in some settings but were associated with higher healthcare costs and sustainability concerns. In Taiwan, a programme targeting 86,000 high-risk individuals reduced CKD-related emergency visits but increased overall healthcare costs (D9).

##### Interprofessional management models

Interprofessional models showed heterogeneous effects depending on professional roles and implementation context.

###### Pharmacist-led interventions

Pharmacist-led interventions produced mixed results. Randomised studies in the USA showed no effect on blood pressure, statin use, screening, or CKD coding (I83, I77). In contrast, a Spanish feasibility study reported identification of renal dosing errors in 8% of prescriptions, generally accepted by GPs, although time constraints limited feasibility (D44). In Canada, pharmacist interventions reduced cardiovascular risk in patients with CKD, particularly in rural areas (I79), while combined pharmacist–NP involvement in the USA improved access to nephrology without reducing costs (D46).

###### Role of nephrology-trained nurse practitioners in supporting primary care

Nephrology-trained nurse practitioners were associated with improvements in CKD management. A New Zealand randomised trial showed better control of progression risk factors and self-care through NP–PCP collaboration (I82). In England, a large interventional study improved CKD identification, proteinuria testing, and blood pressure targets, without affecting eGFR (I70).

###### Support from nephrologists and tele-expertise

Nephrologist support was associated with changes in coordination and referral practices. In the USA, risk-stratified nephrology co-management *via* EHRs reduced CKD progression in stage 3a patients (D43). GPs reported improved access to specialist advice following nephrologist-led training in Canada (D42), while networking initiatives in Italy improved testing and identification (I85). E-consultation reduced face-to-face referrals and increased GP confidence in the USA (I89), and Dutch teleconsultation improved referral appropriateness without clear effects on quality or costs (D36, I73).

##### Tools to support primary healthcare professionals

Several organisational and digital tools were evaluated, with heterogeneous reported effects.

###### Identification through coding and CKD registers

Coding improved albuminuria testing but had little impact on prescribing or blood pressure control (D28, D20). Large cohort studies in the USA and UK showed that CKD identification was associated with better guideline-based prescribing (D7, D5). However, randomised trials of CKD registers yielded inconsistent results (I75, I76), and qualitative data highlighted misclassification issues related to single eGFR values (Q61).

###### Pay-for-performance schemes based on quality indicators

Financial incentives influenced practice. In the UK, inclusion of CKD in the Quality and Outcomes Framework was associated with increased GP awareness and improved disease control (D45), and was perceived as more effective than guidelines alone (Q61). Nevertheless, gaps persisted in Canada regarding medication management, patient education, and referral coordination (D8, D25).

###### Clinical decision support and care coordination tools

Decision-support tools were associated with improved diagnosis and referral processes. Canada’s CKD Pathway increased urine testing with modest prescribing effects (Q66, D6). Alert-based systems improved perceived patient safety without increasing referrals (Q59), and joint GP–nephrologist toolkits enhanced CKD identification and co-management (Q68).

##### Patient education interventions

Patient education interventions were associated with improvements in several outcomes across studies. Lifestyle and educational programmes slowed eGFR decline and improved continuity of care (I74), enhanced medication adherence and kidney outcomes in patients with diabetes (I80), and improved blood pressure, quality of life, and patient–provider communication (I90, Q48). Similar benefits were observed in Taiwan and England, including improved self-efficacy, blood pressure control, and reduced healthcare costs (I88, I78).

##### Multimodal interventions combining human support and tools

Multimodal interventions showed more consistent positive effects across studies. US studies combining clinical decision support with practice facilitation improved CKD coding, screening, referrals, and kidney outcomes (I72, D2, I71). Combined lifestyle and pharmacological interventions reduced blood pressure, albuminuria, and HbA1c in New Zealand (D3, D45), while an interprofessional care pathway improved cardiovascular risk management in the Netherlands (I81).

## Discussion

This integrative review describes the management of patients with early-to-moderate CKD in primary care in high-income countries, highlighting differences between guideline recommendations and reported practices. PCPs face multiple challenges in screening at-risk populations, identifying CKD, and implementing guideline-based kidney- protective care. CKD is often perceived rather as a cardiovascular risk factor or a natural consequence of ageing and these ideas hinder recommended management and patient empowerment [121].

A previous review [122] assessing barriers and facilitators to CKD diagnosis and management in primary care identified major obstacles as time constraints, discomfort in disclosing a CKD diagnosis, and dissatisfaction with the existing guidelines, whereas digital tools and collaborative care were considered as key facilitators. Two systematic reviews reported beneficial effects of interprofessional care for patients with stage 3–5 CKD [123,124]. Our review highlights limited coordination and co-management with nephrologists. One qualitative study found that the availability of multiple urine testing options created confusion among general practitioners and was associated with potentially inappropriate referrals [125]. Limited albuminuria testing represents not only a screening gap but also a prognostic limitation, as risk stratification tools such as the KDIGO heat map require both eGFR and albuminuria. Albuminuria may be under-recognised because it is typically asymptomatic and can occur despite preserved eGFR, making CKD less visible to both patients and clinicians.

Galbraith et al. evaluated chronic disease management interventions implemented by primary care providers for patients with CKD and reported that few studies were available and that findings were heterogeneous, suggesting that multimodal interventions may be required [126]. In our review, multimodal strategies commonly combined clinical decision support (CDS), multidisciplinary involvement, and educational tools for both clinicians and patients.

A recent systematic review [127] reported that the automated identification of high-risk patients combined with management advice, educational materials for GPs, and remote case review by a nephrologist, was associated with increased guideline-concordant care integrated into routine workflow [128]. The composition of interprofessional teams may need to be adapted to local contexts and could involve task delegation or role enhancement among selected healthcare professionals [129].

The strengths of this study include its comprehensive scope, international perspective, adherence to PRISMA guidelines, and independent appraisal of study quality. Several limitations should also be considered. Difficulty accessing some full-text and the inherent subjectivity of JBI appraisal tools may have influenced study assessment. The inclusion of moderate CKD stages (eGFR as low as 45 ml/min/1.73m^2^), complicated study selection due to inconsistencies in reporting on stage 3 A *vs.* 3B. Furthermore, this integrative evidence synthesis did not aim to assess the effectiveness of interventions or to compare healthcare systems across countries. Most included studies originated from a limited number of countries (USA, UK, Canada, Australia, and the Netherlands), which may limit generalisability. Because studies span more than two decades, findings reflect management practices reported over time rather than a real-time snapshot of current care. Research in low- and middle-income countries would be valuable but currently most do not have access to eGFR or albuminuria testing within primary care settings [130,131].

## Conclusion

Management of early-to-moderate CKD in primary care varies substantially across settings. Multimodal strategies combining organisational support, decision-support tools, interprofessional collaboration, and patient education were most consistently associated with improvements, suggesting promising directions for context-adapted implementation.

## Supplementary Material

Supplemental Material

## Data Availability

All data extracted and synthesised for this review are included in the Supplementary Materials (SD10–SD12). Additional data (search strategies, scoring grids, screening logs) are available from the corresponding author upon reasonable request.

## Protocol registration

PROSPERO International Prospective Register of Systematic Reviews.
