# Supplementary material for: Challenges and strategies for managing early-to-moderate chronic kidney disease in primary care: A systematic integrative review in high-income countries
Source: Eur J Gen Pract. 2026 Aug 5;32(1):2704287. doi: 10.1080/13814788.2026.2704287 (PMC13446063; doi:10.1080/13814788.2026.2704287)
Supplement: Supplementary file 1 [file IGEN_A_2704287_SM3863.docx]

**Title:**. Challenges and strategies for managing early-to-moderate chronic kidney disease in primary care: a systematic integrative review in high-income countries

Supplementary Data

Contents

- Supplementary Table S1. Prisma check-list for this study
- Supplementary Table S2.  List of the 80 high-income countries according to the 2022 world bank atlas
- Supplementary Table S3. Description of Knowledge Translation+ and PDQ-Evidence databases
- Supplementary Table S4. PESICO model and strategy for selecting and excluding keywords, MeSH terms and synonyms for search equations, date of consultation in databases
- Supplementary Table S5. Description and procedure for JBI tool scoring
- Supplementary figure S6. Distribution by study type
- Supplementary figure S7. Geographical distribution of studies
- Supplementary Table S8 : Years of publication of studies
- Supplementary Table S9 : Description of the participants included
- Supplementary Table S10 : Domains of CKD Care Management Addressed by Included Studies
- Supplementary Table S11 : Description and summary of the descriptive studies
- Supplementary Table S12 : Description and summary of the qualitative studies
- Supplementary Table S13 : Description and summary of the interventional studies

**S1. Checklist of PRISMA items.**

Page numbers refer to the manuscript, SD refers to the Supplementary Data

| **Section and Topic** | **Item #** | **Checklist item** | **Location where item is reported** |
| --- | --- | --- | --- |
| **TITLE** | | |  |
| Title | 1 | Identify the report as a systematic review. | Title page, P1 |
| **ABSTRACT** | | |  |
| Abstract | 2 | See PRISMA 2020 for Abstracts checklist. | P1 |
| **INTRODUCTION** | | |  |
| Rationale | 3 | Describe the rationale for the review in the context of the existing knowledge. | P2-3 |
| Objectives | 4 | Provide an explicit statement of the objective(s) or question(s) the review addresses. | P4 |
| **METHODS** | | |  |
| Eligibility criteria | 5 | Specify the inclusion and exclusion criteria for the review and how studies were grouped for the syntheses. | P4, P5 |
| Information sources | 6 | Specify all databases, registers, websites, organisations, reference lists and other sources searched or consulted to identify studies. Specify the date when each source was last searched or consulted. | P5, SD4 |
| Search strategy | 7 | Present the full search strategies for all databases, registers and websites, including any filters and limits used. | SD 4 |
| Selection process | 8 | Specify the methods used to decide whether a study met the inclusion criteria of the review, including how many reviewers screened each record and each report retrieved, whether they worked independently, and if applicable, details of automation tools used in the process. | P6 |
| Data collection process | 9 | Specify the methods used to collect data from reports, including how many reviewers collected data from each report, whether they worked independently, any processes for obtaining or confirming data from study investigators, and if applicable, details of automation tools used in the process. | P6 |
| Data items | 10a | List and define all outcomes for which data were sought. Specify whether all results that were compatible with each outcome domain in each study were sought (e.g. for all measures, time points, analyses), and if not, the methods used to decide which results to collect. | P6 |
|  | 10b | List and define all other variables for which data were sought (e.g. participant and intervention characteristics, funding sources). Describe any assumptions made about any missing or unclear information. | P6 |
| Study risk of bias assessment | 11 | Specify the methods used to assess risk of bias in the included studies, including details of the tool(s) used, how many reviewers assessed each study and whether they worked independently, and if applicable, details of automation tools used in the process. | P6, SD5 |
| Effect measures | 12 | Specify for each outcome the effect measure(s) (e.g. risk ratio, mean difference) used in the synthesis or presentation of results. | N/A |
| Synthesis methods | 13a | Describe the processes used to decide which studies were eligible for each synthesis (e.g. tabulating the study intervention characteristics and comparing against the planned groups for each synthesis (item #5)). | P5 |
|  | 13b | Describe any methods required to prepare the data for presentation or synthesis, such as handling of missing summary statistics, or data conversions. | N/A |
|  | 13c | Describe any methods used to tabulate or visually display results of individual studies and syntheses. | P6, SD10,  SD11, SD12, SD13  Table 1-5 |
|  | 13d | Describe any methods used to synthesize results and provide a rationale for the choice(s). If meta-analysis was performed, describe the model(s), method(s) to identify the presence and extent of statistical heterogeneity, and software package(s) used. | P6 |
|  | 13e | Describe any methods used to explore possible causes of heterogeneity among study results (e.g. subgroup analysis, meta-regression). | N/A |
|  | 13f | Describe any sensitivity analyses conducted to assess robustness of the synthesized results. | N/A |
| Reporting bias assessment | 14 | Describe any methods used to assess risk of bias due to missing results in a synthesis (arising from reporting biases). | P5 +P6 |
| Certainty assessment | 15 | Describe any methods used to assess certainty (or confidence) in the body of evidence for an outcome. | N/A |
| **RESULTS** | | |  |
| Study selection | 16a | Describe the results of the search and selection process, from the number of records identified in the search to the number of studies included in the review, ideally using a flow diagram. | P6 + fig 1 |
|  | 16b | Cite studies that might appear to meet the inclusion criteria, but which were excluded, and explain why they were excluded. | P5 |
| Study characteristics | 17 | Cite each included study and present its characteristics. | SD11, SD12  SD13 |
| Risk of bias in studies | 18 | Present assessments of risk of bias for each included study. | Table 1-3 |
| Results of individual studies | 19 | For all outcomes, present, for each study: (a) summary statistics for each group (where appropriate) and (b) an effect estimate and its precision (e.g. confidence/credible interval), ideally using structured Tables or plots. | N/A |
| Results of syntheses | 20a | For each synthesis, briefly summarise the characteristics and risk of bias among contributing studies. | SD11,  SD12,  SD13 |
|  | 20b | Present results of all statistical syntheses conducted. If meta-analysis was done, present for each the summary estimate and its precision (e.g. confidence/credible interval) and measures of statistical heterogeneity. If comparing groups, describe the direction of the effect. | N/A |
|  | 20c | Present results of all investigations of possible causes of heterogeneity among study results. | N/A |
|  | 20d | Present results of all sensitivity analyses conducted to assess the robustness of the synthesized results. | N/A |
| Reporting biases | 21 | Present assessments of risk of bias due to missing results (arising from reporting biases) for each synthesis assessed. | N/A |
| Certainty of evidence | 22 | Present assessments of certainty (or confidence) in the body of evidence for each outcome assessed. | N/A |
| **DISCUSSION** | | |  |
| Discussion | 23a | Provide a general interpretation of the results in the context of other evidence. | P7-P15; Fig 3 |
|  | 23b | Discuss any limitations of the evidence included in the review. | P16 |
|  | 23c | Discuss any limitations of the review processes used. | P16 |
|  | 23d | Discuss implications of the results for practice, policy, and future research. | P16 |
| **OTHER INFORMATION** | | |  |
| Registration and protocol | 24a | Provide registration information for the review, including register name and registration number, or state that the review was not registered. | P4 |
|  | 24b | Indicate where the review protocol can be accessed, or state that a protocol was not prepared. | P4 |
|  | 24c | Describe and explain any amendments to information provided at registration or in the protocol. | N/A |
| Support | 25 | Describe sources of financial or non-financial support for the review, and the role of the funders or sponsors in the review. | After tables |
| Competing interests | 26 | Declare any competing interests of review authors. | After tables |
| Availability of data, code and other materials | 27 | Report which of the following are publicly available and where they can be found: template data collection forms; data extracted from included studies; data used for all analyses; analytic code; any other materials used in the review. | After tables  SD10, 11,12, 13 |

**S2. List of the 80 high-income countries according to the 2022 world bank atlas**

| [Andorra](https://worldpopulationreview.com/countries/andorra-population) | Gibraltar | [Palau](https://worldpopulationreview.com/countries/palau-population) |
| --- | --- | --- |
| Antigua and Barbuda | [Greece](https://worldpopulationreview.com/countries/greece-population) | [Poland](https://worldpopulationreview.com/countries/poland-population) |
| Aruba | [Greenland](https://worldpopulationreview.com/countries/greenland-population) | [Portugal](https://worldpopulationreview.com/countries/portugal-population) |
| [Australia](https://worldpopulationreview.com/countries/australia-population) | Guam | [Puerto Rico](https://worldpopulationreview.com/countries/puerto-rico-population) |
| [Austria](https://worldpopulationreview.com/countries/austria-population) | [Hong Kong](https://worldpopulationreview.com/countries/hong-kong-population) | [Qatar](https://worldpopulationreview.com/countries/qatar-population) |
| [Bahamas](https://worldpopulationreview.com/countries/bahamas-population) | [Hungary](https://worldpopulationreview.com/countries/hungary-population) | Saint Kitts and Nevis |
| [Bahrain](https://worldpopulationreview.com/countries/bahrain-population) | [Iceland](https://worldpopulationreview.com/countries/iceland-population) | Saint Martin |
| [Barbados](https://worldpopulationreview.com/countries/barbados-population) | [Ireland](https://worldpopulationreview.com/countries/ireland-population) | [San Marino](https://worldpopulationreview.com/countries/san-marino-population) |
| [Belgium](https://worldpopulationreview.com/countries/belgium-population) | Isle of Man | Saudi Arabia |
| Bermuda | [Israel](https://worldpopulationreview.com/countries/israel-population) | [Seychelles](https://worldpopulationreview.com/countries/seychelles-population) |
| British Virgin Islands | [Italy](https://worldpopulationreview.com/countries/italy-population) | [Singapore](https://worldpopulationreview.com/countries/singapore-population) |
| [Brunei](https://worldpopulationreview.com/countries/brunei-population) | [Japan](https://worldpopulationreview.com/countries/japan-population) | Sint Maarten |
| [Canada](https://worldpopulationreview.com/countries/canada-population) | [Kuwait](https://worldpopulationreview.com/countries/kuwait-population) | [Slovakia](https://worldpopulationreview.com/countries/slovakia-population) |
| Cayman Islands | [Latvia](https://worldpopulationreview.com/countries/latvia-population) | [Slovenia](https://worldpopulationreview.com/countries/slovenia-population) |
| Channel Islands | [Liechtenstein](https://worldpopulationreview.com/countries/liechtenstein-population) | [South Korea](https://worldpopulationreview.com/countries/south-korea-population) |
| [Chile](https://worldpopulationreview.com/countries/chile-population) | [Lithuania](https://worldpopulationreview.com/countries/lithuania-population) | [Spain](https://worldpopulationreview.com/countries/spain-population) |
| [Croatia](https://worldpopulationreview.com/countries/croatia-population) | [Luxembourg](https://worldpopulationreview.com/countries/luxembourg-population) | [Sweden](https://worldpopulationreview.com/countries/sweden-population) |
| Curacao | Macau | [Switzerland](https://worldpopulationreview.com/countries/switzerland-population) |
| [Cyprus](https://worldpopulationreview.com/countries/cyprus-population) | [Malta](https://worldpopulationreview.com/countries/malta-population) | [Taiwan](https://worldpopulationreview.com/countries/taiwan-population) |
| [Czech Republic](https://worldpopulationreview.com/countries/czech-republic-population) | [Monaco](https://worldpopulationreview.com/countries/monaco-population) | Trinidad and Tobago |
| [Denmark](https://worldpopulationreview.com/countries/denmark-population) | [Nauru](https://worldpopulationreview.com/countries/nauru-population) | Turks and Caicos Islands |
| [Estonia](https://worldpopulationreview.com/countries/estonia-population) | [Netherlan](https://worldpopulationreview.com/countries/netherlands-population)ds | [United Arab Emirates](https://worldpopulationreview.com/countries/united-arab-emirates-population) |
| Faroe Islands | New Caledonia | [United Kingdom](https://worldpopulationreview.com/countries/united-kingdom-population) |
| [Finland](https://worldpopulationreview.com/countries/finland-population) | [New Zealand](https://worldpopulationreview.com/countries/new-zealand-population) | United States |
| [France](https://worldpopulationreview.com/countries/france-population) | Northern Mariana Islands | United States Virgin Islands |
| French [Polynesia](https://worldpopulationreview.com/continents/polynesia-population) | [Norway](https://worldpopulationreview.com/countries/norway-population) | [Uruguay](https://worldpopulationreview.com/countries/uruguay-population) |
| [Germany](https://worldpopulationreview.com/countries/germany-population) | [Oman](https://worldpopulationreview.com/countries/oman-population) |  |

**S3 Description of the Knowledge Translation + and PDQ-EVIDENCE databases:**

Knowledge Translation + or KT+ is a service provided by the McMaster University's Health Information Research Unit. It provides access to current ‘T2’* knowledge translation evidence (i.e. research on the gap between knowledge and practice), including original published articles and systematic reviews on healthcare quality improvement, health services research and patient adherence.)

PDQ-Evidence , “pretty darn quick”-Evidence facilitates rapid access to the best available evidence for decision-making about healthcare systems. It includes systematic reviews, general review summaries (including evidence-based guidance notes), primary studies included in systematic reviews and structured summaries of this evidence.

**S4. PESICO Model and selection/exclusion strategy for keywords, MeSH terms and synonyms for search equations**

PESICO Model:

| P | Participants | Patients > 18 early-to-moderate stage CKD  Excluding pregnancy, advanced CKD, end-stage, dialysis, transplant  Excluding acute kidney failure |
| --- | --- | --- |
| Environment | Environment | Outpatient care settings (non-hospitalised care) |
| Stakeholders | Stakeholders | Healthcare professionals: primary care (GPs, emergency physicians, nurses, nurse practitioners (NPs), pharmacists)  secondary and tertiary (nephrologist, cardiologist, diabetologist)  Excluding biologists, excluding caregivers |
| I | Intervention | Management (identification, management and referral in the care pathway)  Excluding laboratory-based screening studies not addressing clinical management. |
| C | Intervention used for comparison | Recommendations for management |
| O | ~~Outcomes (judgement criteria)~~  Outcome domain | ~~Describe the management in theory and practice~~ Empirical descriptions of CKD care management in primary care across six domains*: screening and detection, CKD identification and coding, kidney-protective management and follow-up, referral and collaboration with nephrology, patient education and self-management, and organisation of care pathways. |

NB a narrative review of the best practice recommendations on the management of early to moderate stage CKD patients is the subject of a specific publication.

* *The six broad domains presented here were used to guide study identification and selection and to organise extraction of heterogeneous evidence. They served as descriptive mapping categories rather than predefined analytical themes. Themes and subthemes presented in the Results emerged inductively during the integrative narrative synthesis process.*

**Main keywords with their MESH equivalents, possible synonyms, as well as excluded terms**

- Early-to-moderate chronic kidney disease

| **MESH term** | **MESH Synonyms** |
| --- | --- |
| renal insufficiency/cies , chronic | kidney insufficiencies/cy , chronic |
| chronic renal disease/ses | chronic kidney disease/ses |
| early stage |  |
| Moderate stage |  |

Excluding :

| kidney failure, chronic | chronic renal failure  end stage kidney disease  end-stage kidney disease  end stage renal disease  end-stage renal disease  end stage renal failure  end-stage renal failure |
| --- | --- |
| dialysis/ses |  |
| kidney transplantation | grafting, kidney  kidney grafting  kidney transplantations  renal transplantation  renal transplantations  transplantation, kidney  transplantation, renal  transplantations, kidney  transplantations, renal |

- Management and Care pathway

| **MESH term** | **MESH Synonyms** |
| --- | --- |
| critical path/way/s/ | Clinical pathway |
| gatekeeping | gatekeeper |
| Case management | Management, case |
| Patient care management | Management, patient care  Care management, patient |

Excluding :

| Biological assay | Bioassays, biologic assay... |
| --- | --- |
| Model of care | Nursing model |

- Guidelines

| **MESH term** | **MESH Synonyms** |
| --- | --- |
| Guideline/  practice guidelines | Clinical guidelines |

- Protagonists : ambulatory patients, primary care, general practitioners, emergency physicians, geriatricians, nurses, advanced practice nurses, nephrologists, diabetologists, cardiologists, angiologists excluding biologists and care givers

| **MESH term** | **MESH Synonyms** |
| --- | --- |
| outpatients | Out patient, out patients, out-patient, out-patients |
| primary health care | care, primary  care, primary health  health care, primary  healthcare, primary  primary care  primary healthcare |
| general practitioners | Family practitioner, physician, general practice physician  general practice physicians  general practitioner  physician, general practice  physicians, general practice  practice physicians, general  practitioner, general  practitioners, general |
| emergency medicine | Medicine, emergency |
| nurses | nurse |
| nurse practitioners | nurse practitioner  practitioner, nurse  practitioners, nurse |
| nephrologists | nephrologist |
| cardiologists | cardiologist |
| endocrinologists | endocrinologist |

Excluding :

| caregivers | Carer, care givers, Family caregivers |
| --- | --- |
| biologist | biology |

**Strategy for developing research equations in several databases**

**PubMed :** procedure for elaborating the equations on 2022/03/13

Filters applied: From 2000/1/1 to 2022/9/01, Humans, English, Adult: 19+ years.

#1 : Chronic renal insufficiency

#2 : Chronic kidney Failure

#3 : Dialysis

\# 4 : critical pathway

#5 : gatekeeping

#6 : case management

#7 : primary care

MeSH Terms Results

#1. chronic renal insufficiency 48254

#1 NOT (#2 OR #3) 11657

[#1 NOT (#2 OR #3)] AND #4 : 6

[#4 OR #5 OR #6] : 4807

[#1 NOT (#2 OR #3)] AND [#4 OR #5 OR #6] : 8

#1 NOT (#2 OR #3)] AND [#4 OR #5 OR #6 OR #7] : 263

**Embase** procedure for elaborating the equations on 2022/03/29

Emtree : results

#1 : Chronic kidney failure : 179370

#2 : #1 AND filters : 52621

- publication year 2000-2022

- publication types : article, article in press, preprint, review), study types filter (case control study, clinical article, clinical trial, cohort analysis, comparative study, controlled study, cross sectional study, longitudinal study, human study, multicenter study, observational study, practice guideline, prospective study, randomized controlled trial topic, retrospective study, systematic review

- age : adults > 18

- diseases : Chronic kidney failure, kidney disease, kidney dysfunction, kidney failure

#3 Renal replacement therapy OR Dialysis OR end stage renal disease : 384187

#4 : #2 NOT #3 : 31075

#5 : clinical pathway OR patient referral OR 'case management' OR 'patient care' OR 'primary medical care' : 671703

#6: #4 AND #5 :1141

**PDQ evidence =** procedure for elaborating the equations on 2022/03/01

The advanced search engine of this database allows the following equation to be developed: Full query: (title:((title:(chronic kidney disease) OR abstract:(chronic kidney disease)) AND (title:(care pathways) OR abstract:(care pathways)) OR (title:(critical pathways) OR abstract:(critical pathways)) OR (title:(patient care management) OR abstract:(patient care management))) OR abstract:((title:(chronic kidney disease) OR abstract:(chronic kidney disease)) AND (title:(care pathways) OR abstract:(care pathways)) OR (title:(critical pathways) OR abstract:(critical pathways)) OR (title:(patient care management) OR abstract:(patient care management))))

- 172 results

**KT+** procedure for elaborating the equations on 2022/03/01

Searching by keywords "chronic kidney insufficiencies" the result is 21 shortlisted items out of 118 in total, 4 of which seem to match the inclusion criteria on the title

**S5. Description and procedure for scoring with JBI tools**

The Joanna Briggs Institute (JBI) offers a series of critical appraisal tools to help researchers and healthcare professionals assess the methodological quality of scientific studies. In this review we used the tools for:

- analytical cross-sectional studies: checklist for Analytical Cross Sectional Studies.

- case-control studies: checklist for Case Control Studies.

- cohort studies: checklist for Cohort Studies.

- prevalence studies: checklist for Prevalence Studies.

- qualitative studies: checklist for Qualitative Research.

- checklist for Quasi-Experimental Studies.

- hecklist for Randomized Controlled Trials.

These tools are designed to assess the reliability, relevance and results of published articles, helping to identify potential biases and determine the validity of conclusions.

Each evaluation grid comprised between 8 and 13 items. For each item:

- When the answer was yes, the evaluator counted 1 point.

- When the answer was no, no points were awarded.

- When the item was not applicable for methodological reasons, it was deducted from the total so as not to unfairly devalue the final mark.

- When the answer to an item was unclear or incomplete (‘unclear’) in the text of the article, the assessor counted 0.5 points.

**Figure S6. Distribution by study type**

**Figure S7. Geographical distribution of studies**

**Figure S8. Year of publication of studies**

**S9. Summary of participants included in the studies**

|  | Descriptive | Qualitative | Interventional | Total |
| --- | --- | --- | --- | --- |
| **Number of patients** | 33.395.008 | 146 | 75.772 | 33.470.926 |
| **Number of GPs** | 2268 | 440 | 274 | 2688 |
| **Number of nurses/NPs** | 50 | 68 | 234 | 352 |
| **Number of nephrologists** | 7 | 49 | 4 | 60 |
| **Number of pharmacists** |  | 4 |  | 4 |
| **Number of interns** |  | 6 | 56 | 62 |
| **Number of endocrinologists** |  | 4 |  | 4 |
| **Number of social workers** |  | 4 |  | 4 |
| **Number of dieticians** |  | 3 |  | 3 |
| **Number of coordinators** |  | 1 |  | 1 |
| **Number of Public Health Doctors** |  | 2 |  | 2 |

**S10 – Domains of CKD Care Management Addressed by Included Studies**

These domains were used as a descriptive framework for mapping extracted findings and should not be interpreted as predefined analytical themes.

| **Domain of CKD care management** | **Descriptive studies** | **Qualitative studies** | **Intervention studies** |
| --- | --- | --- | --- |
| **Screening and detection of CKD in at-risk populations** | D7, D9, D18, D22, D24, D27 | Q52 | I87 |
| **Identification, diagnosis and coding of CKD** | D5, D19, D20, D28, D31, D33, D34, D35 | Q50 | I75, I76, I86 |
| **Kidney-protective clinical management and follow-up**  **(risk-factor control, prescribing, monitoring)** | D3, D4, D8, D10, D11, D12, D15, D16, D17, D25, D26, D38, D39, D41, D44, D45 | Q47, Q54, Q60, Q63, Q69 | I70, I72, I77, I79, I80, I83 |
| **Referral and collaboration with nephrology** | D1, D13, D14, D21, D23, D29, D30, D32, D36, D37, D42, D43, D46 | Q55, Q56, Q64, Q68 | I73, I81 |
| **Patient education and self-management** | — | Q48, Q49, Q51, Q53, Q65 | I74, I78, I82, I88, I90 |
| **Organisation of care pathways,**  **decision support and**  **health system interventions** | D2, D6, D40 | Q57, Q58, Q59, Q61, Q62, Q66, Q67 | I71, I84, I85, I89 |

**S11. Summary of the descriptive studies**

Supplementary Tables S11–S13 provide detailed extracted data for each included study, including study objectives, participant characteristics and main findings. Corresponding study codes, authors, publication years and reference numbers are presented in Tables 1–3 of the main manuscript.

| Citations | Objectives | Population under study | Main results |
| --- | --- | --- | --- |
| Van Dipten *et al.*,  2017  D1 | Identify patient characteristics and quality of care received by CKD patients for whom nephrologist referral recommendations were not followed. | 47 practices for 2772 patients  eGFR : 48 to 45 | Patient characteristics: generally older, less cardiovascular disease (CVD)  Quality of care for patients treated in primary care but not referred despite indication:  - More advanced CKD, not properly monitored  - Less labelled CKD  - Poor control of potassium, BP and metabolic parameters |
| Fox *et al.*,  2008  D2 | Evaluate a multimodal intervention (practice facilitators; OADC; monthly training) to raise physicians' awareness of CKD and KDOQI recommendations. | 2 public health practices (6 GPs and 4 APNs)  81patients with eGFR <60ml/min/1.73m²  The APNs created the CKD patient register and set up the electronic system | Identification of CKD improved significantly, from 21% to 79%  Diagnosis of anaemia increased significantly, from 33% to 67%  Reduction in the use of NSAIDs and metformin  Mean eGFR increased from 45 to 47 ml/min/1.73m2 |
| Tan *et al.*,  2014  D3 | Evaluate a community-based program to improve BP in T2-CDM patients  Intervention: progressive escalation of antihypertensive therapies to achieve and maintain BP < or equal to 125/80 mmHg  1st line: ACE inhibitor or ARB2  2nd line: + Thiazide diuretic  3rd line: + Calcium antagonist  Promotion of medication compliance  Education on lifestyle, diet and personal care | 47 patients (43 complete data) with T2DM and CKD.  No distinction between stages 3a and 3b but 77.5% of patients were stage 0 to 3. | Significant reduction in BP thanks to intensification of anti-HTA treatment and promotion of compliance with MS medication  Reduction in albuminuria and Hba1c. |
| Danforth *et al.*,  2019  D4 | Identify factors influencing follow-up of a first abnormal eGFR | Retrospective analysis  244 550 patients eGFR < 60 ml/min/1.73m²   mean eGFR 51 to 54 ml/min/1.73m² | 58% of patients did not have a suiTable biological check-up after their 1st abnormal eGFR (97% checked too late or not checked at all)  UnsuiTable biological follow-up more frequent in patients with no comorbidity, female, young, Caucasian or in physicians who are late with their messages  SuiTable biological follow-up more frequent if automatic alerts are present in the record, prescription by a specialist physician and in patients consulting their results online  Quantitative and qualitative results suggest ways to improve follow-up of laboratory results by involving more clinical staff and reporting abnormal eGFR results. + |
| Jain *et al.*,  2014  D5 | Determine the prevalence of CKD stages 3-5 and compare the quality of care of CKD-identified patients with that of non-identified patients. | 426 primary care centres (2.7 million patients included).  Based on anonymous records from the THIN database  (The Heath Improvement Network)  Majority of patients stage 3a | Between 2005 and 2009: CKD 3 to 5 prevalence increased from 0.3% to 3.9%.  In 2009, 30,440 CKD patients not enrolled in a CKD registry and 60,705 patients enrolled without biochemical CKD criteria  Enrolment in a registry is associated with higher age, male sex, diabetes, hypertension, CVD and higher CKD stage.  Patients with unidentified CKD compared with misidentified patients were less likely to have a BP measurement (90% *vs.* 97%) or to reach BP targets (47% vs. 51%) or recommended PU (12% *vs.* 20%). |
| Donald *et al.*,  2021  D6 | Determine whether an online care pathway for CKD is associated with improvements in CKD management including ACR testing and guideline-recommended drug therapy.  CKD-CP | Adults aged 18 or over with mean eGFR < 60 mL/min/1.73m2: cohort of 345,058 adults, 67% of whom were stage 3a. | After implementing the clinical pathway:  - Slightly higher and statistically significant rates of ACRs in the Calgary area ( > in individuals without diabetes)  - Slight improvements in guideline-compliant medical management (RASi/statin prescribing) but no significant improvement  Online clinical pathway = innovative approach to widespread dissemination of guidelines, interesting on a population scale, no change in rural areas |
| Bansal *et al.*  2020  D7 | Determine CKD screening and recognition rates, the factors associated with these rates, and assess the effect of CKD awareness on care delivery. | Cohort of 270170 veterans with hypertension and/or diabetes mellitus  62% dfg > 45 | 94% of patients had either an eGFR or urine protein assay, or both.  56% Urine protein assay, 35% if HTA isolated  36% biological signs of CKD, 44% of which were recognized (code and/or nephrological referral).  CKD due to ACR alone less recognized (19%) compared to eGFR alone (44%) or both (67%).  Recognition high in non-white races and increased with worsening of eGFR and ACR.  If CKD detected and recognized, prescription of RASi, statins and diuretics higher, *vs.* NSAIDs lower. |
| Nash *et al.*,  2017  D8 | Use recently established public health quality indicators for CKD to determine the proportion of patients receiving recommended renal care | 3 cohorts of patients aged 40 or over (a total of 410,409 patients):   (1) eGFR screening cohort (patients with an initial eGFR value < 60 ml/min/1.73m2): 223,994 patients, 83.8% with eGFR between 45 and 59 (2) RAC screening cohort (patients   with RAC > 3 mg/mmol): 132,442 patients including 89% with a known eGFR value (93.5% > 45 ml/min/1.73m2)  (3) CKD cohort (patients with 2 eGFR values < 60 ml/min/1.73m2 between 3 and 18 months apart): 184,557 patients including 65.1% stage 3a | Recommendations not always followed in current practice.  Screening and recognition of CKD: less than 50% of patients had a follow-up test after a first abnormal eGFR to confirm the presence of CKD  Renal function monitoring: RAC less frequent than creatinemia  Prescribing indicators: a majority of patients with CKD receive a RASi but room for improvement for statins  Poor monitoring of creatinemia or potassium when a RASi is introduced. |
| Chen, Y. *et al.*,  2020  D9 | Evaluate the effectiveness of the CKD early detection program on patient follow-up | 86581 patients who benefited from the early detection programme for CKD compared with 86581 patients who did not.  Stage 1–3a | Patient participation in the programme:  - Increased the number of examinations and tests prescribed to patients.  - Improved day-to-day monitoring of patients  Expenditure on medical consultations and drugs increased whereas hospital admissions and emergency room visits decreased.  Approximately 50% diabetic patients have annual CKD screening |
| Bramlage *et al.*,  2021  D10 | Compare clinical practice with recommendations to identify areas for improvement in the detection and treatment of CKD | 134,395 DT1 or DT2 patients  Mean eGFR of 92.7 mL/min/1.73 m2 in the DT1 group and 68.3 mL/min/ 1.73 m2 in the DT2 group | Environ 50% patient diabétique a un dépistage annuel de la CKD  Less than 50% of patients achieve a target HbA1c of <7.0%  Recommended use of SGLT-2i and GLP1-RA is well below 10%  HTA controlled in 66%  RASi are used in approximately 50% of patients. |
| James *et al.*,  2022  D11 | Identify patient characteristics and complications according to each stage of CKD | 123,807 patients with eGFR between 5 and 75 mL/min/1.73m² mean eGFR between 61 and 65 mL/min/1.73m² depending on the cohort. | No improvement in ACR screening after KDIGO recommendations in 2012 (<10% of patients)  Alongside the decrease in eGFR and increase in albuminuria, there was an increased risk of CV events, AKI and all-cause mortality |
| Chong *et al.*,  2022  D12 | Determine whether poor continuity of care is associated with higher rates of acute care and lower prescribing of guideline-recommended drugs | Cohorte de 86 475 patients atteints de CKD stade 3-4 (60% stade 3a) | Patients with poor continuity of care had 1.5 times the risk of all-cause hospitalisation and an emergency department visit.  Poor continuity of care was associated with lower rates of prescription of recommended drugs |
| Ghimire *et al.*,  2022  D13 | Evaluate patterns of referral of patients with CKD to nephrology, and differences in characteristics and outcomes between patients referred according to guidelines and patients referred without following guidelines | Cohort of 69,372 patients who visited a nephrologist; 56% stage 1-3a patients. | The overall trend towards referral to nephrologists increased between 2006 and 2019 with only 41% of referrals in line with the recommendations: a large number of patients suffer from mild CKD and may be more suiTable for monitoring by GPs. |
| Singh *et al.*,  2017  D14 | Examine the feasibility of adhering to the *Kidney Disease: Improving Global Outcomes* (KDIGO) 2012 recommendations for nephrology referral | 56,461 primary care patients | 5593 (9.9%) had CKD, 2851 of whom required specialist advice, and only 21% saw a nephrologist in 2013  If the recommendations had been followed, there would have been a 38% increase in the total volume of nephrology patients and a 67% increase in new patients.  Adherence to the referral recommendations seems difficult given the existing nephrology offer, especially as the population studied benefits from good access to care. |
| Kampmann *et al.*,  2022  D15 | Quantify the prevalence of statin use before and after the publication of the KDIGO guideline (2013). | A total of 19 743 patients with a mean eGFR < 60 ml/min/1.73 m2, aged 50 or over and non-dialys for all the periods under evaluation. | No change in the prevalence of statin use up to 2 years after publication of the KDIGO guidelines. |
| Van Gelder *et al.*,  2016  D16 | Evaluate the quality of CKD care in primary care according to patient characteristics and practices in order to adapt care. | 8794 patients  Mean eGFR: 52 ml/min | Complete monitoring of CKD in 42% of CKD patients.  Metabolic parameters monitored in 2% of patients.  Target BP achieved in 43.1% of patients.  FPs documented CKD in 31.4% of CKD patients. |
| Jones *et al.*,  2013  D17 | Evaluate the care of patients with CKD and diabetes. | 2 710 patients with an eGFR < 60 ml/min and at least one year of biological follow-up data  Mean eGFR ml/min (± SD) : 47.5 ± 9.1 | Nephrology wards see a minoritý of diabetic patients with CKD.  Among them, 70% had no signs of CKD, whereas 50% of patients cared for exclusively by the GP and diabetologist had a decline in eGFR of >5 ml/min/year.  Patients treated in nephrology were younger than those treated by FPs or diabetologists.  A large proportion of patients with sTable renal function were followed by a nephrologist, whereas a large proportion of patients with progressive CKD were not sufficiently referred to a nephrologist. |
| Perkins *et al.*  2016  D18 | Evaluate trends in the management of early-stage CKD (screening, treatment, referral to nephrology) and their associations with clinical outcomes. | Stage 3 CKD:  728 in 2004-2006,  788 in 2007-2009  956 in 2010-2012  eGFR 49-50 | Significant increase over time  - of PU screening (x2)  - prescription of statins  - referral to nephrology  have not changed:  - RASi prescription rates  - adjusted mortality,  - progression of CKD  - Cardiovascular hospitalisations  PU dosage remains low - of 50% CKD population in 2010-2012 |
| Weckmann *et al.*,  2022  D19 | Evaluate the management and quality of care by evaluating how CKD was identified in Primary Care, knowledge of the diagnosis by the patient, the frequency of monitoring referral to nephrologists. | . 1778 patients with clinical data from the Study of Health in Pomerania cohort (SHIP-START)  10% of participants with reduced eGFR (<60 ml/min/1.73m2), the majority of whom were stage 3a. | Patient identification increased with the eGFR stage (G3a 20%, G3b 61%, G4 75%, G5 100%): more reliable in the higher stages.  AU rarely leads to coding for chronic kidney disease.  Inadequate AU assessment if eGFR is impaired.  Frequency of tests partially higher than recommended (lack of care coordination).  Patients were unaware of their renal function. |
| Frigaard *et al.*,  2019  D20 | Evaluate the relevance of the eGFR diagnosis of CKD and assess the link between CKD coding and medical management | 2214 patients with two eGFRs between 15 and 59 ml/min/1.73m² at least 90 days apart  mean eGFR 52 ml/min/1.73m² (73% stage 3a) | Diagnosing CKD by eGFR alone overestimated the prevalence of the disease: of the 50 patients randomly selected, only 34 (68%) had CKD confirmed by the nephrologist  44% of the patients included had CKD recorded in the electronic medical record.  The presence of CKD coding in the patient record was associated with higher rates of UA testing and statin use, but not with RASi use. |
| Van Dipten *et al.*,  2021  D21 | Assess the characteristics and number of patients who could potentially be referred to primary care, using predefined criteria developed by nephrologists and GPs. | 300 patients included: 102 patients from VieCurie Medical Center (VMC), 100 patients from Canisius Wilhelmina Hospital (CWH) and 98 patients from Radboud university medical center (RUMC).  eGFR ranged from stage 1 to 5 but the mean eGFR was 42 ml/min. | 78 of the 300 patients (26%) in the consultations met the criteria for referral to PH, with a variation of 23% to 30% per center.  Of these patients :  - 56.4% were men, median age 70  - Lower mean frequency of monitoring (3.0 vs. 4.1/year) and mean eGFR 46 vs. 40 ml/min/1.73m2.  - More often affected by heart failure (19.2% vs. 7.8%) and transient ischemic attack or stroke (21.8% vs. 10.8%)  - Less often affected by cancer (16.9% *vs.* 20.8%).  - All stages of CKD: stage 1 (6.4%), stage 2 (14.1%), stage 3a (21.7%), stage 3b (35.9%) and stage 4 (14.1%). |
| Alfego *et al.*  2021  D22 | Evaluate the frequency of CKD screening tests in patients at risk that conformed to the recommendations. | 28 million patients coded as having diabetes mellitus and/or high blood pressure or according to their prescriptions. | 16.2% diabetes 63.8% hypertension and 20.1% both.  - 80.3% not assessed in accordance with guidelines  - 21.0% have ACR compared with 89.6% eGFR.  - complete tests at least once 28.7% diabetes, 10.5% hypertension and 41.4% both.  State by state variation but national rate increased modestly from 2013 and 2018.  Absence of CKD coding (4.8% of individuals at low risk, 10.1% at moderate risk, 42.3% at high risk and 55.5% at very high risk according to the KDIGO classification.)  Majority of eGFR and CAR tests requested by MS *vs.* CPR by nephrologists |
| Chu *et al.*,  2022  D23 | Evaluate the link between CKD and referral to a nephrologist | 291,155 CKD patients (eGFR < 60 ml/min/1.73m² ou ACR > 30 mg/g)  75% stage 1 to 3a patients | No ACR done in 59% of cases  Only 55% of patients meeting the criteria were referred to a nephrologist (64% for eGFR < 30 ml/min/1.73m²; 51% for RAC > 300 mg/g)  In the multivariate models, more severe UA was systematically associated with a higher probability of nephrological care in each eGFR category  Results overestimated because study carried out on a population with access to care. |
| Manski *et al.*,  2018  D24 | To investigate: (1) the proportion of T2DM people visiting an Australian FP who were screened or followed up for CKD; (2) the proportion of T2DM with no documented diagnosis of CKD who present with CKD; and (3) factors associated with screening and recording a diagnosis of CKD for T2DM people in Australian GP practices. | 90550 T2 diabetic patients seen in 495 GP practices | 44,394 (49.0%) had appropriate screening or follow-up.  8030 (8.9%) patients were diagnosed with CKD, 6597 (7.3%) patients were not diagnosed with CKD despite having proven CKD.  Older age and hypertension or dyslipidaemia were associated with increased likelihood of CKD registration.  Older patients, hypertension or dyslipidaemia and open medical records more frequently + probability of being appropriately screened.  Screening and follow-up for CKD appear to be sub-optimal. |
| Bello *et al.*,  2019  D25 | Evaluate the current state of CKD management using quality indicators in Canadian public health practices to identify gaps in care and guide the development and implementation of national quality improvement initiatives. | Cohort of 46,162 patients with stage 3 to 5 CKD (a total of 24,368 (68.6%) patients had stage 3a CKD with a mean eGFR value of 47.7 mL/min/1.73m2) | 4 out of 12 quality indicators were successfully achieved by 75% or more of the cohort: renal function testing and monitoring, BP management, glycaemic control.  As the stages progressed, care was increasingly in line with guidelines, except for stage 5.  Areas requiring improvement: detection and recognition of CVD risk associated with CVD (albuminuria), use of recommended drugs, monitoring of renal function after prescription of RASi. |
| Bezabhe *et al.*,  2020  D26 | Examine the quality of prescriptions in public health for patients with CKD | 44,259 patients with stage 3 to 5 CKD (57.8% with eGFR of 45 to 59 ml/min/1.73m2) | Gaps identified in the prescription of nephro-protective drugs (RASi and statins) and avoidance of nephrotoxic drugs (NSAIDs, metformin, digoxin).  Patients in disadvantaged or remote areas more at risk.  60.2% were identified as CKD with eGFR + albU |
| Hagnas *et al.* 2020  D27 | Estimate the prevalence of CKD among patients with Type 2 diabetes in primary care in Finland, and evaluate CKD screening and the proportion of patients receiving hypoglycemiant medication and drugs to prevent cardiovascular disease. | 5 112 patients with Type 2 diabetes at a single health center in Rovaniemi | 30.1% were CKD. Of these, 19.6% had moderate albU and 3.2% had severe albU.  57.0% received i RAS.  eGFR, CRA and UA not measured in 21.9%, 53.6%, 41.2% respectively  Screening by two recommended measures (eGFR and albuminuria) was not sufficiently performed in T2DM. |
| Samal *et al.*, 2014  D28 | Evaluate the link between CKD coding in the electronic file and the quality of care | 3149 patients with stage 3 or 4 CKD Mean eGFR 45 ml/min/1.73m² | Only 16% of stage 3 or 4 patients had CKD documented in their records  Coding was positively associated with severity of CKD, male gender and black and Hispanic patients  Coding favoured monitoring by eGFR measurement (97 vs 94%) and proteinuria/albuminuria measurement (47 vs 40%)  But did not lead to better BP or RASi prescription (after adjustment for severity of CKD, gender and ethnicity) |
| Samal *et al.*,  2015  D29 | Evaluate the quality of care of stage 3-4 CKD patients by PHPs alone *vs.* co-management nephrologist | 3 118 patients with stage 3-4 chronic renal insufficiency (62% 3a, 30% 3b and 8% 4) including 11 % who were managed by a nephrologist.    Analyses divided between stages 3 and 4 | Co-management associated with younger age, male sex, minority ethnicity (black; Hispanic), hypertension, diabetes  After adjustment, co-management was positively associated with eGFR testing (98 vs 94%), UA (82 vs 36%), RASi prescription for stage 3 only (79 vs 69%), monitoring of anaemia and metabolic bone disease  Co-management not associated with control of CV risk factors: lipids, control of BP |
| Koraishy *et al.*, 2017  D30 | Calculate the probabilities of referral to nephrology according to race/ethnic origin and rate of progression of CKD | 2,170 patients  (75% eGFR patients > 60 ml/min/1.73m², 17% stage 3a, 8% stage 3b or 4) | Referral to nephrologist more frequent in patients with rapid eGFR decline *vs.* slow decline, and in black patients vs. white patients.  Rapid decline more frequent in patients who are black, elderly, living in precarious conditions, or with hypertension, T2DM or obesity.  After adjustment, no ethnic disparity found despite lower eGFR in black patients at time of referral. |
| McIntyre *et al.*,  2012  D31 | To describe follow-up needs and assess knowledge of the diagnosis in the cohort of patients with stage 3 CKD. | 32 general practices, 1741 patients  Stage 3 (23% stage 3b), mean eGFR 52.5 ± 10.4 ml/min/1.73 m2. | 2/3 of stage 3 patients required treatment: - BP control or reduction in antihypertensive drugs  - Treatment of mild anaemia and 8.2% received advice on exploring anaemia.  - For 7.5% of participants, cessation of nephrotoxic drugs.  Only 6% needed to be referred to a nephrologist.  41% were unaware of their kidney disease.  Identification of factors favouring knowledge of CKD: age < 75, high level of education, stage 3b CKD and high level of albuminuria. |
| Schulz *et al.*,  2022  D32 | To examine the profile of French patients consulting a nephrologist for the first time and compare these characteristics with the KDIGO, HAS and Canadian kidney failure risk equation (KFRE) recommendations. | 10 nephrologists  Free referrals: at the request of the FP, the patient or another specialist, with no triage process.  Classification of two groups of patients: Group 1  (GFR ≥ 30 and PCR < 0.5) and group 2 (GFR<30or PCR ≥ 0.5)  eGFR:  - 47% between 45 and 60 ml/min/1.73m2)  - 33% between stages 1 and 2  - 19% at stage 4  - 4% at stage 5 | Nephro-angiosclerosis was the most frequently reported for advanced stages versus stage 1 glomerulopathy.  43% of patients who saw a nephrologist according to HAS recommendations did not need one.  58% of patients who saw a nephrologist according to KDIGO recommendations did not need one.  20% of patients who saw a nephrologist according to KFRE recommendations did not need one. |
| Gaffney *et al.*,  2014  D33 | Evaluate the factors (demographic, clinical and psychological) associated with self-reporting of chronic kidney disease by patients | 436 patients with stage 3 CKD from 24 GP practices  Stage 3 (a + b) with 75% 3a  Questionnaire implemented 6 to 8 weeks post-consultation with a GP | Approximately 50% of patients did not self-report CKD.  Multivariate analysis revealed that female patients, patients with stage 3b CKD and more anxious patients were more likely to report having CKD. |
| Greer *et al.*,  2011  D34 | Identify the characteristics and determinants leading to discussion of CKD in at-risk patients, in Public Health consultations. | Interviews of 236 hypertensive patients with 40 GPs conducted in parallel with a randomized trial designed to improve BP control | Discussion of CKD present in 26% of consultations: content focused on laboratory tests (89%), treatment of DRFs (28%), and causes (26%) of CKD  CKD discussion less frequent in patients with university education or few comorbidities, by doctors with > 10 years' experience  CKD discussion more frequent if visits are long or if diabetes is discussed  38% of patients correctly screened had CKD, but only 4 reported having it; no difference in the occurrence of CKD between healthy patients and those with CKD. |
| Burke *et al.*, 2014  D35 | Determine the understanding of CKD in patients referred to an outpatient nephrology clinic for the first time. | 217 patients interviewed using questionnaires  Stages of patients interviewed: 0 (8%), 1 (24%), 2 (11%), 3 (38.5%), 4 (18%), 5(0.5%) | 82% of patients referred by their GP.  Poor knowledge of CKD: 35.8% reported no education, 46.2% some but not adequate, 18% found education prior to referral adequate.  Common misunderstandings and misconceptions about CKD.  Recognition, education and treatment of CKD need to be improved in Public Health. |
| Scherpbier-de Haan *et al.*,  2013  D36 | Evaluate nephrology teleconsultation on patient referral rates and examine the ease of use of teleconsultation (investment in time, implementation in daily work and nephrologist response time). | 122 tele-nephrology consultations carried out by 28 FP practices and 5 outpatient nephrology departments.  eGFR 46 mL/min/1.73 m2 | In the absence of a tele-nephrology consultation, 43 patients (35.3%) would have been referred by their GP, whereas the nephrologist considered it necessary to refer only 17 patients (13.9%).  The GP would have treated 79 patients in primary care without referral to the nephrologist, whereas the nephrologist considered it necessary to see 10 of these patients in consultation.  The time commitment per consultation was less than 10 minutes.  Consultations took place mainly during office hours.  The response time was 1.6 days.  Most consultations concerned eGFR, proteinuria and BP. |
| Meran *et al.*,  2011  D37 | Evaluate the follow-up and changes in biological parameters of patients referred to primary care in order to determine whether there is an increased risk of referring the patient to primary care according to current recommendations. | 88 patients included in the analysis from a nephrology referral at the NHS Trust hospital in Southeast Wales.  Over 60% in stage 3  23% in stages 1, 2 or normal 17% in stage 4. | Before referral, 93% of patients had a sTable eGFR, 77.5% had a blood pressure below the threshold (140/90) and 97.7% had a haemoglobin level above 10 g/dl.  Following referral, 83% of patients had their eGFR recorded by their GP and 92.6% were measured within appropriate timeframes. The majoritý of patients (82%) had their eGFR improve or stabilise after reorientation and only three patients had a significant fall in eGFR. |
| Tatematsu *et al.*,  2022  D38 | To compare the results of two surveys (2013 and 2022) in order to analyse changes in practice in the medical management of CKD by GPs. | 2921 GPs | The frequency of biological examinations, the use of eGFR and the use of RASi as reno-protectors were significantly higher in 2019 than in 2013.    Medical cooperation for referral to the nephrologist according to the eGFR measurement during the medical consultation increased.  More FPs expressed dissatisfaction with the advice or explanations given to CKD patients or elements of CKD treatment by nephrologists. |
| Tatematsu *et al.*,  2021  D39 | Assess the current state of medical management of CKD and evaluate the use of CKD recommendations by GPs. | 585 GPs (including 171 who use the recommendations and 414 who do not). | 84% of GPs were aware that CKD guidelines had been revised  - 76 % envisaged using an RASi  Urinary protein tests: 65.5% measured proteinuria, 20% assessed albuminuria  Use of therapies to limit chronic anaemia: 78% used EPO but the target Hb objectives were correct for 27.5% (Hb greater than 11 g/dL)  Nephroprotective treatments:  - 76% considered using an RASi  - 47% considered using them permanently for their renoprotective effects |
| Abdel Kader *et al.*,  2014  D40 | Understanding beliefs and barriers to optimal management of non-diabetic CKD | 165 médecins via un questionnaire en ligne  56% internistes, 39% GPs | 51% of doctors said they were unfamiliar with the KDIGO/KDOQI recommendations  75% said they screened non-diabetic hypertensive patients for ABR, regardless of eGFR  16% did not consider isolated severe albuminuria to be a marker of CKD  For some doctors, ABR is an onerous (30%) and unreliable (14%) procedure  Doctors found RASi useful, but paradoxically more so in cases of moderate than severe albuminuria. |
| Seidu *et al.*,  2022  D41 | To assess the knowledge and skills of healthcare professionals involved in the management of CKD in people with diabetes in Europe (Turkey, Spain, the United Kingdom, Italy, Belgium, Sweden, the Netherlands, France, Greece, Portugal and Hungary), Denmark and other countries). | 266 professionals: general practitioners (82.7%), nurses (9.4%).  No eGFR because studies focused on healthcare professionals.  Introduction of an online questionnaire sent to professionals | 1/3 are fully confident in the screening and diagnosis of CKD in people with diabetes.  Slightly more than 1/4 fully confident about the presentation, staging and prognosis of CKD but 11% not sure how to predict the prognosis of CKD using the recommendations.  1/3 confident, 1/4 fully confident, no need for aids to manage complications of CKD in diabetics.  1/3 fully confident in preventing or slowing the progression of CKD, on the use of RASi and iGLP1 and SGLT2  For all aspects of management, most respondents were confident, but needed to learn more or needed additional support. |
| Pang *et al.*  2016  D42 | Evaluate a pilot nephrologist training/support programme for PCPs on:  - overall uptake by PCPs,  - predictors of uptake,  - changes in PCPs' knowledge and comfort levels of CKD care,  - ease of access and  - satisfaction with nephrologist support, and PCPs' overall experience of the programme. | 89 PCPs mentored by 7 nephrologists | No predictor of increased interactions between mentees was identified but increase in number of practice years (> 5 ≤ 5 years) associated + frequency of interactions and mentees with - of pt CKD tend to use the programme more  Self-assessed knowledge and comfort levels with all CKD-related topics increased significantly (screening, identification, management).  Ease of access to a nephrologist increased for all modes of communication measured with the exception of webinar or videoconferencing. Mentor diaries showed that individual case consultations were most often by mail.  Overall positive experience: 68% “very useful” or “extremely useful” 44% “always applicable” to their practice, and 53% extremely likely to recommend the programme to a colleague or friend, 87% would use it again. |
| Lee *et al.*  2012  D43 | Evaluate the effect of proactive population-based nephrological monitoring (PPNO) on the progression of CKD | 2958 case/control pairs with CKD 3a (cases after PPNO controls before)  PPNO: a model to risk stratify a population with CKD and which provides unsolicited nephrological consultations by GPs through an integrated electronic medical record (EHR).  Nephrologists collaborate with their primary care colleagues, helping them to manage the patients they can manage and to refer those who need specialist care. | Progression was less severe over the post-PPNO period. (+2.6 % remaining in Stage 3a). Progression from 3a to 3b < 2.2 % from 3a to 4/5 < 0.2 % and from 3a to terminal kidney failure < 0.24 % |
| Escribá-Martí *et al.*,  2022  D44 | Identify and refer patients to the GP with low eGFR who are using nephrotoxic drugs or who may require dose adjustment. | 198 patients, recruited from 4 pharmacies  635 medicines were analysed  85% were stages 1-3a | 7.9% of drugs had their efficacy affected by renal function: dose adjustment or discontinuation required.  The FP accepted the recommendations for 14 drugs (i.e. 2.3% of the drugs used): dose adjustment for 6 and discontinuation for 8.  Out of 20 changes proposed at stage 3a (0 at stage 1-2): 3 molecules /13 had their dosage adjusted (levetiracetam for 86-year-olds and metformin and sitagliptin for 60-year-olds) and 5 molecules /7 were discontinued (3 NSAIDs, 1 beta-blocker and 1 metformin).  Medication reconciliation lasted an average of 23 minutes. |
| Jameson *et al.*,  2014  D 45 | Estimate the prevalence of CKD in the UK in 2010 and evaluate the prevalence, comorbidities and drug prescriptions associated with the evolution of CKD according to the 2006 good practice recommandations. | Use of data from the General Practice Research Database (GPRD), which has been collecting anonymized data from over 400 primary care practices in the UK since 1988.  78.4% stage 1 to 2 14% stage 3a, 7.6% stage 3b, 4 and 5  Introduction of recommendations on CKD in the QOF (Quality and Outcomes Framework) in 2006. | Diagnoses of cardiovascular disease-related comorbidities (hypertension, hypercholesterolemia, diabetes and CVD), and treatment with antihypertensive and lipid-lowering drugs, increase as CKD worsens.  CKD is sTable or progressive, with more patients improving their disease stage and reducing their comorbidities through the use of antihypertensive and lipid-lowering treatments.  Introducing recommendations in the QOF has raised awareness of CKD among UK doctors, leading to earlier and better control of CKD progression. |
| Smith *et al.*,  2016  D46 | Evaluate the effect of a public health model integrating GP, nephrologists, APN and pharmacists specialized in nephrology, on identifying CKD, referral to a nephrologist and the associated care costs. | 1,255 stage 3 to 5 CKD patients with comorbidities  Stage 3a 66%, 3b 24% and 4-5 10%    APNs and pharmacists made recommendations on prescriptions and APNs also on referral to the nephrologist for each CKD patient. | Increased use of RAC testing and eGFR calculation in the whole cohort (+ 121% and + 73%, respectively) and in patients with comorbidities.  The proportion of patients with eGFR < 60 ml/min/1.73m2 increased significantly from 2.2% to 3.8% in the entire cohort, from 6.3% to 12.7% in diabetic patients, from 5.6% to 9.9% in hypertensive patients and from 10.8% to 23.7% in heart failure patients. |

**S12. Summary of qualitative studies**

Supplementary Tables S11–S13 provide detailed extracted data for each included study, including study objectives, participant characteristics and main findings. Corresponding study codes, authors, publication years and reference numbers are presented in Tables 1–3 of the main manuscript.

| Citations | Objectives / experiment | Population under study / data collection and analysis | Résultats principaux |
| --- | --- | --- | --- |
| Vest *et al.*,  2015  Q47 | Analyse the implementation of CKD recommendations by PHPs | 24 GPs  3 SN/PH medical assistants  Semi-directed individual interview,  thematic content analysis  + classification according to the theory of the normalisation process | Lack of knowledge of recommendations by GPs (lack of ongoing training, frequent changes in recommendations)  Difficulties in appropriating new technologies and the volume of data, impossibility of creating registers on a specific pathology, work overload.  Lack of auditing, feedback on practices.  Lack of patient education, limited insurance reimbursements for treatments and biological tests, transportation difficulties. |
| Havas *et al.*,  2017  Q48 | Examine patients' experiences and perceptions of a CKD self-management intervention  Programme self managment support: CKD-SMS = 12-week intervention delivered in individual face-to-face and telephone formats to 66 people with CKD | 63 patients surveyed, participants in the CKD-SMS program, 63.7% in CKD stages 1, 2 or 3a  semi-structured individual interviews  thematic content analysis | An individualized program such as CKD-SMS provides an opportunity to support patients in effectively managing their health.  Positive effect of the intervention on knowledge about kidneys and CKD.  Strengthened confidence to engage in self-management behaviours and interactions with healthcare professionals. |
| Kahn *et al.*,  2015  Q49 | Understanding the constraints and self-management strategies of low-income patients in CKD | 33 patients (26 stage 3 CKD, 7 stage 4)  from 2 primary care practice centers in East Side, Buffalo, a low-income community.  Semi-structured interviews  Analysis of thematic contents + theory on the process of normalization | Barriers to self-management:  Lack of patient knowledge/explanation of CKD  Asymptomatic nature  Illness rarely considered a separate disease  Time and transport constraints  Inadequate medical coverage  Self-management strategies:  Efforts to modify diet and physical activity  Help from family and church  Use of pillboxes |
| Van Dipten *et al.*,  2018  Q50 | To explore early to moderate CKD patients' knowledge, thoughts and experiences about their CKD and the information provided to them | 21 patients interviewed  eGFR: early to moderate stage, 20 in our target group.  Semi-structured interviews  Constant comparison analysis + Atlas software. | Filling patients' knowledge gaps with misconceptions and half-truths about the causes, symptoms and treatment of CKD.  Anxiety about CKD at the time of diagnosis and a perceived discrepancy with the absence of CKD symptoms following diagnosis.  Physicians minimize the severity of CKD.  No link made between lifestyle and cardiovascular disease on the one hand, and CKD on the other.  Not well informed about the possible consequences of CKD. |
| Hwang *et al.*,  2020  Q51 | To describe the facilitating and limiting factors in the self-management of CKD by pre-dialysis patients | 20 stage 3 patients (90%) and stage 4 (10%)  Semi-directed interviews  Anchored theory analysis and N Vivo software | Facilitators:  - Patient trust and satisfaction with physician  - Family support.  Obstacles :  - Lack of knowledge and awareness of CKD  - Passive attitude towards self-management  - Insufficient communication with doctor (reluctance of patients to ask their doctor about their condition).  - Information overload and too little advice on how to manage their disease, particularly with regard to dietary recommendations.  - Patients worry about being a financial and social burden on their families.  Improve patient-doctor communication:  - workshops and home visits  - Disseminate accurate information online  - Strengthen patient involvement and optimize family support by involving family members in care.  - Clear dietetic recommendations and specific advice |
| Sinclair *et al.*,  2017  Q52 | Identify barriers and facilitators to CKD screening practices by GP nurses | 26 SRNs  Online interview 8 open questions  Analysis via the theory of planned behavior | Gap between SRNs' understanding of the benefits of screening and actually doing it  Facilitators: early identification would enable early management and minimize progression of CKD; ideal place of the SRN  Obstacles: lack of time, lack of knowledge, risk of causing stress for the patient, interprofessional conflict |
| Greer *et al.*,  2012  Q53 | Identify barriers to CKD patient education by primary care professionals (PCPs) | 15 doctors and 3 nurses,  3 group interviews  Continuous comparison analysis Grounded theory (GT)  27 GPs | Barriers:  Patients' lack of awareness and understanding of their CKD  PHPs did not consider CKD as a distinct disease, lacked knowledge and feared burdening their patients with this diagnosis  Time constraint and lack of dedicated remuneration  Lack of educational resources to pass on to patients |
| Van Dipten *et al.*,  2018  Q54 | Understand the views of GPs on the management of early-stage CKD patients and the applicability of the national interdisciplinary guideline. | 27 GPs  4 group interviews  Grounded theory | CKD perceived as a difficult, abstract concept with no fixed definition: disease per se or a cardiovascular risk factor.  Insufficient knowledge of the clinical consequences of CKD, and of treatment, with doubts expressed about the real benefit of following the recommendations.  Main concern was to inform patients without creating anxiety. Lack of materials to help inform patients.  No consensus on delegation of tasks to APNs  No consensus on the benefits of involving nephrologists. Feeling of loss of control following referral to a nephrologist. Not enough concertation  Suggests considering the patient's social, economic and historical context, not just the biological data, and giving more advice on how to empower patients with doubts about the benefit/time ratio for doing so. |
| Lo *et al.*,  2016  Q55 | Examine the perceptions of primary and tertiary healthcare professionals regarding the key factors influencing optimal healthcare for patients with diabetes and CKD | 65 professionals: 22 GPs, 35 tertiary care professionals (endicrinologists, nephrologists, diabetes or nephro APNs, nurse educators, dieticians, social workers and 8 diabetes and kidney service managers  Group interviews: 4 with GPs; 6 with tertiary care professionals  8 semi-structured individual interviews with diabetes and kidney service specialists  Constant comparison + generic approach  Patton and Harding. | 4 key factors identified as requiring improvement:  1. Self-management  2. Access to tertiary care  3. Care coordination  4. Reactive approach to care (*vs.* preventive)  1 key factor identified by unit head physicians: Need for + quality improvement activities and feedback on performance. |
| Greer *et al.*,  2019  Q56 | Identify barriers perceived by PHPs to co-management of CKD with nephrologists | 19 interns, 13 GPs  4 discussion groups  Inductive content analysis by conceptual category + Atlas software | Obstacles:  Reports unavailable or lacking in detail (treatments introduced, management to be carried out, responsibilities of each doctor)  Limited access to nephrologists (consultations, advice)  Improve EMR interoperability for efficient receipt of reports, better defined care plans  Better knowledge of recommendations by PHPs for relevant referrals and clarification of the reason for referral. |
| Blakeman *et al.*,  2012  Q57 | Identify the processes underlying the management of CRM in primary care. | 11 GPs and 10 nurses  Semi-directive interviews  TPN Analysis | PCPs were concerned that the announcement would create anxietý in patients, particularly the elderly and those in stage 3a CKD, for whom the clinical benefit was considered less certain. Reluctance to use the label ‘CKD’, need to ‘play down’ the disease to avoid frightening patients.  To avoid talking about ‘disease’, some professionals presented renal function as a marker of well-being or vascular risk, but there was no overall consideration of the patient (as a chronic kidney patient) during vascular follow-up appointments.  It may be helpful to talk about CKD as a marker of "well-being", as efforts are focused on improving cardiovascular health in general.  To reduce patient anxiety: - Announcement should take place during consultations with public health professionals  - Team approach to reduce anxietý related to CKD management. |
| Gulla *et al.*,  2017  Q58 | Identify physicians' needs in terms of clinical decision support systems for CKD | 12 Public Health doctors  semi-structured individual interviews  continuous comparison, grounded theory | To improve the system, PHPs would like it to incorporate easily accessible CKD recommendations and better visualization of eGFR evolution  For CKD follow-up, alerts on non-control of risk factors (BP, diabetes) or the presence of complications associated with CKD (anemia, hyperkalemia, metabolic bone disease)  For proper referral, better visualization of previous nephrology visits, nephrologist availability or a tool prioritizing patients most at risk of complications could prove useful Physicians expressed unease about the responsibilities of each doctor and the appropriateness of referring certain elderly patients to a nephrologist. |
| Thomas *et al.*,  2019  Q59 | Identify practitioners' perceptions of the use of the eGFR estimation tool, a computer tool that alerts patients in the event of a fall in eGFR: new eGFR value <60mL/min/1.73m2 or a decrease of greater than or equal to 10mL/min/1.73m2 compared with the previous measurement. | 6 GPs, 1 pharmacist, 1 coordinator  Semi-structured individual interview  Thematic analysis, framework model | Well-organized practices with strong administrative support found the tool easily integrated and expressed greater motivation to use it. The tool was perceived as promoting patient safetý and was used́ for learning about CKD management, both at individual and practice level. |
| Crinson *et al.*  2010  Q60 | Understand practitioners' views on chronic kidney disease and its management. | 26 GPs, 9 APNs and 1 pharmacist  5 group interviews  Thematic analysis, framework model | Not all professionals have the same vision of CKD, and not all have adopted the guide. Some sought to implement the guide in its entirety, while others limited themselves to the pay-for-performance objectives.  Almost all practitioners had doubts about whether CKD was really a disease; they questioned the diagnosis of CKD based solely on eGFR; they wondered whether CKD in the elderly was part of natural aging; and they had found it difficult to explain CKD to patients without frightening them.  Problems and skepticism regarding BP goals and recognized gaps in education. |
| Nihat *et al.*  2016  Q61 | Explore the extent to which professionals in participating practices were aware of the QUICK intervention and why a change in practice may have occurred.  Chronic kidney disease quality improvement study (QICKD), comparing audit-based education (ABE) and the provision of guidelines and clinical cases (G&C) with usual practice, to improve systolic BP control in MS | Between 72 and 117 primary care practice professionals: GPs, APNs, medical assistants and coordinators.  12 group interviews with 6-9 professionals  Thematic analysis, framework model | Need to improve accuracy and understanding of CKD registry (inclusion without 2 eGFRs at least 3 months apart).  Difficult for some to announce CKD with patients, who need to be reassured. Some avoided using the term.  Practitioners were more motivated to change their practice by the pay-for-performance indicators than by the evidence-based guidelines  The study helped develop practical advice, and the presence of a doctor or nurse specialized in nephrology was more useful than the information available.  Participants could see how prompts, role-playing in virtual consultations, workshops and updates could be useful tools to improve their understanding of CKD, but the concept of CKD, how risk is stratified and management changes were a challenge to explain to patients. |
| McBride *et al.*,  2014  Q62 | Explore PCPs' attitudes and beliefs about the benefits or otherwise of a CKD registry versus other chronic disease registries  Identify the key enablers to the successful implementation of a CKD Registry in secure primary care. | 19 GPs (11 medical center directors, 9 quality improvement managers)  1 APN  Semi-directed individual interview  Grounded theory analysis | PCPs in the San Francisco network believe that a CKD registry has the potential to improve CKD patient care, particularly if it  - identifies CKD patients who no longer have access to care  - recognizes CKD progression early,  - tracks CKD quality measures,  - provides alerts for abnormal lab results  - makes available decision support in the form of evidence-based algorithms and referral strategies.  Facilitator: system redesign, + expansion of non-medical staff roles in patient care, with priority given to team care and feedback on performance... |
| Simmonds *et al.*  2016  Q63 | Examine and understand the different views and experiences of Public Health professionals in identifying and monitoring early-stage CKD compared with current guidelines, particularly at stage 3. | 25 clinicians: 16 GPs, 3 APNs, 4 nephrology consultants, 2 public health physicians.  Semi-directed individual interview  Thematic analysis + continuous GT comparison | Two related general themes of dissonance and consonance in PHP perspectives on early-stage CKD monitoring.  GP: initial discomfort at being prompted QOF to register and monitor stage 3 CKD patients, as this contradicted existing beliefs about the meaning and status of mildly reduced renal function.  The inclusion of the words “chronic” and “disease” make CKD a source of tension as too simplistic to describe a medical concept that is primarily linked to an increased risk of cardiovascular morbidity.  They consider that classification guidelines oversimplify a complex picture of interrelated health and demographic factors, posing ethical dilemmas in diagnosis and disclosure (tensions around disclosing a potentially frightening diagnosis to patients).  All agreed on the need to better understand renal decline and increase proteinuria testing to reduce overdiagnosis and identify patients at risk of progression who would benefit from early intervention.  Clinicians recommended a holistic approach to CKD patients, who represent a barometer of overall health. |
| Haase *et al.*  2019  Q64 | Nephrologists' views on the interface and GPs management of non-dialysis CKD patients 20 nephrologists | 20 nephrologists  Semi-directive interview  Analysis by grounded theory | Optimization of associated diseases, adaptation of medications and patient awareness of CKD are the main measures in managing CKD.  Opinions differ as to the responsibility for lifestyle interventions, patient education and timing of referral: Most for referral of patients at risk of progression with co-management some preferred referral of all patients with early CKD and some that their care should be entirely by nephrologists.  Their doctor-patient communication is mainly rooted in the framework of medical and scientific knowledge, without taking into account the patient's point of view or shared decision-making to prioritize patients' health goals.  Most see patient and PCP education as their role. |
| Rainey *et al.*,  2020  Q65 | Understanding the experience of people with CKD 1 to 3b invited to individual or group education sessions | 9 patients 3 had group education 6 individual  Semi-directed individual interview  Thematic analysis | a. Recognition of CKD unexpected if asymptomatic  b. Motivation to change. Although shocking, motivation to change lifestyle. relief at knowing that they could do something to reduce their risk of worsening CKD (hypertension, diabetes)  c. Dependence on experts: difficult to monitor CKD via blood tests, depended on professionals to interpret results. Most people were convinced that their CKD was being monitored, but not always informed of progress. |
| Donald *et al.*,  2016  Q66 | Describe the development and implementation of an online CKD care pathway: CKD-CP and obtain users' perspectives and experiences to monitor the use and feasibility of this approach  CKD-CP = interactive online tool to assist providers in 3 aspects of managing CKD: diagnosis, management and referral | 10 GPs, 3 nephrologists, 2 pharmaciens, 3 IDE 10 FPs, 3 nephrologists, 2 pharmacists, 3 SRNs  Individual interviews  Thematic content analysis | Online care pathway identified as preferred approach to facilitate CKD-CP and reduce the gap between evidence and practice.  Evaluation of the tool:  Improved communication within the PCPs team and between PCP and specialists  Could improve work efficiency, Improved confidence  Results suggest that CKD-CP is feasible. |
| Lo *et al.*,  2016  Q67 | Study the FPs' point of view on the obstacles related to the health service in managing diabetes and CKD. | 4 group interviews with general practitioners 6 group interviews with specialists in the tertiary sector + 8 interviews with managers of diabetes and kidney disease treatment units.  4 interviews. Collectives  Thematic analysis Patton and Harding. | Significant proportion of patients with diabetes and CKD cared for by GPs, but lack of knowledge and awareness.  3 main obstacles:  Gaps in treatment and management, insufficient screening for complications  Difficult access to specialized services: (waiting times)  Poor communication and coordination between providers, unclear roles, inconsistent skills and knowledge of GPs. |
| Haley *et al.*,  2015  Q68 | Evaluate the impact of a toolkit developed by the Renal Physicians Association to help PHPs identify CKD and improve communication and co-management with nephrologists  GPs with documents on CKD, a model letter for referral  Nephrologist with a model letter detailing management and respective responsibilities | 9 PHP et 5 cabinets de néphrologie  interviews individuels semi dirigé avant / après expérimentation  Analyse GT | Remarkable improvement by PHPs in identification of CKD, referral and communication / co-management with nephrologists.  Nephrologists improved on referral assistance and communication  Post-intervention interviews showed earlier referral, more frequent screening and greater knowledge of recommendations and risk factors among PHPs  PHPs and nephrologists reported greater satisfaction with their co-management |
| Sperati *et al.*,  2019  Q69 | Identify FPs' perceived barriers to optimal CKD management | 32 Public health professionals  4 group interviews of 8  Thematic analysis | Barriers:  Patients: lack of awareness of CKD, lack of adherence to treatment, cost of care, asymptomatic nature  HCPs: lack of knowledge about CKD and its recommendations, difficulties in monitoring risk factors and prioritising co-morbidities, fatalistic attitude  Healthcare system: inflexible electronic medical record, limited time and tools for therapeutic education  Levers:  Decision support: registration of eGFR by all laboratories, protocols integrated into electronic record  Concise recommendations on CKD  Improve medical coverage and financial compensation for care Better educational tools Inexisting advertisisng campaigns or information inexistantes. To be encouraged |

**S13. Summary of interventional studies**

Supplementary Tables S11–S13 provide detailed extracted data for each included study, including study objectives, participant characteristics and main findings. Corresponding study codes, authors, publication years and reference numbers are presented in Tables 1–3 of the main manuscript.

| Citations | Objectives | Population under study / intervention | Main results |
| --- | --- | --- | --- |
| Major  2019  I70 | Evaluate the effect of a nurse-directed CKD management program on the evolution of renal function in patients with CKD (stages 3 to 5). | 23,357 patients randomized stratified on GPs practice (69 in total) to receive a structured nurse-led CKD management program with possible nephrologist support (the intervention group) or to continue usual GP-led primary care (the control group).  Stages 3 to 5 (including 76.7% stage 3A) | The intervention did not alter eGFR, however, coding of CKD improved́, as did proteinuria testing and the number of patients achieving BP targets. |
| Sequist  2018  I71 | Evaluate the impact of a clinical decision support system and patient self-management tools vs. standard care | 7,691 stage 3 CKD patients separated into two groups (high- and low-risk) regularly informed by message of the benefits of their treatments, BP, biology.  Average eGFR 51 ml/min/1.73m²  153 PHPs receiving electronic alerts during consultation (IEC status, albuminuria, recommendations for referral to nephrologist) | High-risk patients significantly more likely to be referred to a nephrologist in the intervention group (45% *vs.* 34%)  Low-risk patients significantly more likely to be screened for albuminuria in the intervention group (45% *vs.* 21%)  No improvement in RASi prescription in either group  In secondary endpoints, improvement in phosphorus, vitamin D and PTH screening in both groups (and calcium in the high-risk group) |
| Carroll  2018  I72 | Determine whether a multimodal intervention based on the TRANSLATE model of computerized clinical decision support (CDS) and practice facilitators *vs.* CDS alone, slows eGFR decline in CKD patients | 6,699 stage 3 and 4 CKD patients  Average eGFR 47 ml/min/1.73m²  20 CDS practices: computerized algorithms only  10 CDS + GP practices benefited from a referring doctor, a coordinator, monthly meetings, audits and educational programs. | Promising multimodal intervention with no observed decline in eGFR, compared with an annual decline of 0.98 ml/min/1.73m2 in the control group  Minimum decrease in glycated hemoglobin (HbA1c) in the intervention group, 0.14% increase in the control group  No improvement in RASi prescription, NSAID suppression or CKD coding in the record |
| Van Gelder  2017  I73 | Determine whether a consultation platform enabling tele-nephrology would reduce patient referral rates. | 47 general practices: 2,693 patients completed the study.  Mean eGFR 46.7 ml/min  All participating practices received an overview indicating which of their patients met the criteria for consultation and referral to a nephrologist. | Rates of referral to a nephrologist were 2.3% in the intervention group and 3.0% in the control group, a non-significant difference.  The consultation rate in the intervention group was 6.3% versus 5.0%, not significant.  No difference in quality of care or costs.  The majority of GPs had a positive opinion of tele-nephrology. |
| Yamagata  2016  I74 | Compare the effects of an educational intervention on lifestyle changes in early-stage CKD patients seen by FPs. | CKD patients 1-5 followed in GPs  eGFR average 57 and 59  In total, 2,379 patients (1,195 in group A (standard) and 1,184 in group B (advanced intervention)) aged 40 to 74  2 groups: one with standard care with conference on reco several times a year, + an info letter to the GP about CKD treatment every two months *vs.* the same + dietician consultation on weight and ttt, info sent to patients (letter + reminder of appointment 7 days before by mail or phone) and info sent to FPs of these patients (reco, deviation for each patient and info on follow-up, behavioral problems and important points). | The rate of discontinuous clinic visits was significantly lower in group B (16.2% in group A vs. 11.5% in group B, p=0.01). Significantly higher referral and co-treatment rates were observed in group B (p<0.01). The mean rate of eGFR deterioration tended to be lower in group B (group A: 2.6±5.8 ml/min/1.73 m2/year, group B: 2.4±5.1 ml/min/1.73 m2/year, p = 0.07). A significant difference in the rate of eGFR deterioration was observed in subjects with stage 3 CKD (Group A: 2.4±5.9 ml/min/1.73 m2/year, Group B: 1.9±4.4 ml/min/1.73 m2/year, p = 0.03). |
| Drawz  2012  I75 | Evaluate the impact of PHP access to a CKD patient registry and training *vs.* training alone | 781 CKD stage 3 and 4 patients  Average eGFR 48 ml/min/1.73m²  70 PHPs: 56 interns, 8 physicians and 6 APNs  CKD patient registry including RASi, BP, eGFR, CAR, Calcium, Phosphorus, parathyroid hormone (PTH) | In both groups, an increase in PTH measurement (taken as the main criterion, as it is fairly specific to CKD, but not widely prescribed), but no increase in the secondary criteria studied (phosphorus, hemoglobin, RASi, BP control, ACR screening)  The multimodal combination of training and access to a CKD registry showed a significant increase in PTH (from 13% to 28%) compared with the control group (16% to 23%)  However, only 5 of the 37 PHPs in the intervention group accessed the registry during the study. |
| Tuot  2018  I76 | Evaluate the impact of PHP’s access to an CKD patient registry *vs.* routine care | 746 patients with an eGFR between 15 and 59 ml/min/1.73m² or moderate to severe albuminuria (42% stage 1-2, 38% 3a, 15% 3b, 5% 4)  96 PHPs (83% physicians, 17% APN)  Register reminding recommendations and indicating patients without controlled BP, RASi or albuminuria | Primary endpoint at 12 months: no decrease in BP  Secondary endpoints at 12 months: increase in albuminuria tests (Odd-Ratio 2.44) and RASi prescription (82% *vs.* 68% Odds Ratio 2.25) in the intervention group, but not on eGFR and UA levels |
| Peralta  2020  I77 | Evaluate the impact on BP of an Electronic Decision Support (EDS) *vs.*an EDS+ pharmacist call *vs.* routine care | 524 stage 3 CKD patients   Mean eGFR 56 ml/min/1.73m² | No reduction in BP in the intervention group, nor in the prescription of RASis or statins Non-significant increase in coding of CKD in the medical record in the intervention group  74% of physicians with CDS used it  Biases linked to the small number of patients with a confirmed diagnosis of CKD completing the study (few patients contacted by telephone in particular) |
| Blakeman  2014  I78 | Determine the effectiveness of providing CKD information materials versus usual care for stage 3 CKD patients. | 436 Stage 3 CKD patients recruited from 24 GP’s practices in the Greater Manchester area.  75.7% stage 3a, remainder stage 3b  Instauration of a kidney function guide, booklet and interactive website for CKD advice, and telephone support from a non-medical professional. | Improved average quality of life.  Better control of BP.  Reduced health costs.  No change in perception of life. |
| Al Hamarneh  2017  I79 | Demonstrate the effectiveness of pharmacist interventions on the cardiovascular risk in CKD patients | CKD < 60 dfg or UA positive +1 risk factor not balanced.  Mean eGFR 65  2 groups 145: 1 routine care vs.  1 cardiovascular risk treatment assessment and clinical signs by trained pharmacists and CKD pathways (1 consultation per month for 3 months : Therapeutic Patient Education tools on treatment recommendations and adaptation of prescription(s) and/or initiation were made if necessary to achieve the objectives of lipid control, BP, glycemia, smoking cessation, + GP communication | 290 CKD of the 723 participants enrolled in RxEACH. After adjusting for baseline values, the difference in CV risk variation was 20% (- 0.2 mmol/L LDL , - 10.5 mm Hg systolic BP, - 0.7% HbA1c or glycated haemoglobin) and + 19.6% in smoking cessation were observed when comparing the intervention and control groups.  The reduction in cardiovascular risk was greater in rural than in urban areas. |
| Roddy  2022  I80 | Ancillary analysis of a trial of a telephone intervention in T2DM subjects, assessing glycemic control and secondarily renal function. | 271 T2DM patients received daily personalized text messages for 12 months on adherence to treatment, diet and exercise  mean eGFR 77 ml/min/1.73m². | Although the original study showed a reduction in HbA1c at 6 months (but not significantly at 12 months), it did not show any significant improvement in eGFR at 12 months and that initial hyperfiltration may be present in diabetic nephropathy, which may partly distort the results.  There was nevertheless a significant improvement in eGFR compared with the control group for patients with moderate to severe albuminuria at baseline  It should be noted that 76% of patients had an eGFR > 60 ml/min/1.73m2 and 72% had no albuminuria initially, so were less at risk of a rapid decline in eGFR; and initial hyperfiltration may be present |
| Scherpbier-de Haan  2013  I81 | To evaluate the effect of a multi-professional care pathway (training of professionals, structured care by APNs and the possibility of seeking advice from a nephrology team) on the management of CKD patients who also suffer from diabetes or hypertension. | Five practices used the shared care model and four practices followed the usual care for one year, i.e. 90 patients in the intervention group and 74 patients in the control group.  Mean eGFR: 50 and 49 for the control groupe and interventional group, respectively.  Objective: control of blood pressure. | Significant lowering of blood pressure.  Increase in the use of hypolipidemiants, RASi and vitamin D.  But no change in the rate of cholesterol. |
| Walker  2014  I82 | To evaluate an intervention based on self-management in MS, led by a nephrology APN to prevent the progression of CKD in high-risk patients.  Intervention: series of sessions over 12 weeks, led by an APN: assessment + education + planned changes in medication and lifestyle; development of an individual management plan | 2 public health practices, 52 patients (36 of whom completed 12-month follow-up), area of high socioeconomic deprivation.  Patients at a high risk of CKD progression: Type 2 Diabetes Mellitus and uncontrolled hypertension, ACR > 30, history of consultation and poor engagement with the GP.  Mean eGFR : 63 ml/min/1.73m2 | APN ideally positioned for self-management.  Improvement in biomedical outcomes (ACR, eGFR, absolute 5-year cardiovascular risk, blood pressure, glycaemic control)  Improvement in self-management:  Better knowledge of treatments and conditions, compliance and lifestyle (reduction in smoking)  Participation in decision-making, attendance at appointments, monitoring and management of symptoms and early warning signs  Management of effects on acts of daily living and social life |
| Chang  2016  I83 | Evaluate the impact of trained pharmacists on the management of CKD *vs.* standard care. | 47 stage 3a patients with uncontrolled BP   Pharmacists able to monitor BP, start anti-hypertension treatment or statins, prescribe tests to explore proteinuria or lipid anomalies. | No significant improvement in proteinuria screening except for those patients who had not had initial urinary screening   No difference in screening for lipids, prescription of statins and BP control  Small study sample |
| Sinclair  2019  I84 | Evaluate the effectiveness of an e-learning module targeting general practice nurses’ behavioral intentions regarding opportunistic screening practices for people at risk of CKD | 212 SRNs  Active control group (on-line program based on knowledge) and a targeted intervention groupe based on behavior (on-line educational module) | No significant difference between groups in behavioral intention and control during follow-up: on-line targeted behavioral intervention as efficient as an on-line knowledge-based program |
| Pesce  2022  I85 | Evaluate the impact on healthcare professionals of targeted training and networking with nephrologists to raise awareness of CKD, based on the increase in the proportion of patients identified as CKD according to KDIGO in the general population and in patients with diabetes, hypertension and heart failure. | Data extracted from the Millewin digital platforme used by the 17 GPs at the start, i.e. 17 854 patients and after 6 months i.e. 18 662 patients after networking (education, instant messaging and visits) with the 2 nephrologists in the study. | Increase in RAC testing and eGFR calculation in the whole cohort (+ 121% and + 73%, respectively) and in patients with comorbidities.  The proportion of patients with eGFR < 60 ml/min/1.73m2 increased significantly from 2.2% to 3.8% in the whole cohort, from 6.3% to 12.7% in diabetic patients, from 5.6% to 9.9% in hypertensive patients and from 10.8% to 23.7% in IC patients. |
| Akbari  2012  I86 | Evaluate whether the introduction of eGFR by laboratories has had an impact on the number and appropriateness of referrals to nephrologists | 2672 patients included, no eGFR specified for the patients included | Increase in the total number of referrals to the nephrologist by GPs  The proportion of the number of appropriate referrals was identical |
| Vassalotti  2019  I87 | Evaluate the impact of an intervention to raise awareness of CKD screening among at-risk patients in multidisciplinary health units (maisons de santé pluriprofessionnelles=MSPs). | 7 420 patients with high BP /or diabetes  21 healthcare professionals with an initial intervention  - on the interest of early screening and a reminder about the risk factor for CKD  - iconography for CKD staging and recommandations according to the stage | After intervention, 85% eGFR test (unchanged) and 33% ACR (+2%)  No improvement in RASi or statin prescribing  Increased referral of stage 5 patients to nephrologist (from 63% to 83%)  Decreases in hospitalizations and readmissions for the most advanced CKD |
| Chiu Chu L  2013  I88 | Develop a self-management education program and evaluate its effects on self-efficacy, self-management behavior and CKD progression in early-stage patients | 33 patients in south Taïwan  Educational programme towards self-management based on the theory of self-regulation | CKD self-management program resulted in a significant improvement in the self-efficacy of early-stage CKD subjects over a one-year period + a non-significant trend towards improved self-management behavior and improved physiological outcomes in terms of marginally significant reduction in creatinemia and stability of eGFR |
| Stoves  2010  I89 | Evaluate the contribution of a dematerialized electronic medical record-based consultation to improve the primary care pathway for patients with mild-to-moderate CKD | 17 GP’s practices and a nephrology consultation service in Bradford, in the UK.  Patients with low-to-moderate CKD (without details on eGFR) | The dematerialized consultation gave nephrologists access to more clinical information and increased the number of patients referred directly to the GP.  GPs found the service convenient, with useful and timely advice, and that it avoided consultations with nephrologists.  Around half of digital consultations were requests for simple advice, rather than a request for a clinical examination of patients with “borderline” CKD.  Nephrologists' recommendations were well followed, and GPs felt more confident in managing chronic kidney disease. |
| Havas  2018  I90 | Set up and evaluate the Chronic Kidney Disease-Self-Management Support: a theoretical, person-centered self-management intervention for people with stage 1-4 chronic kidney disease. | 77 participants including 49 with stage  1-3a CKD and a 13-week programme (2 face-to-face interviews at weeks 1 and 13 then telephone support as preferred: weekly, twice a month, once a month) | Significant small-to-medium improvements were observed in primary outcomes (self-efficacy: mean difference + 0.8, 95% CI 0.3-1.2, d = 0.4; self-management behavior: mean difference + 6.2, 95% CI 4.5-7.9, d = 0.8). Further significant improvements were observed in secondary outcomes (BP, CKD-specific knowledge, physical activity, fruit and vegeTable consumption, alcohol consumption, health-related quality of life, psychological distress and communication with professionals), with effect sizes ranging from negligible to large (all p < 0.05). |

*Abbreviations : CKD=Chronic Kidney Disease, GP= General Practitioner (family doctor), SRN=State Registered Nurse, ACR=Albumin-Creatinin Ratio, BP=Blood Pressure, T2DM=Type 2 Diabetes Mellitus, RASi=Renin Angiotensin System inhibitors, PHP=Public Health Professional, PTH=Parathyroid hormone, QOF=Quality & Outcomes Framework,*
